# Host‐derived *O*‐glycans inhibit toxigenic conversion by a virulence‐encoding phage in *Vibrio cholerae*

**DOI:** 10.15252/embj.2022111562

**Published:** 2022-12-12

**Authors:** Benjamin X Wang, Julie Takagi, Abigail McShane, Jin Hwan Park, Kazuhiro Aoki, Catherine Griffin, Jennifer Teschler, Giordan Kitts, Giulietta Minzer, Michael Tiemeyer, Rachel Hevey, Fitnat Yildiz, Katharina Ribbeck

**Affiliations:** ^1^ Department of Biological Engineering Massachusetts Institute of Technology Cambridge MA USA; ^2^ Department of Microbiology and Immunology Stanford University Stanford CA USA; ^3^ Department of Biology Massachusetts Institute of Technology Cambridge MA USA; ^4^ Department of Microbiology and Environmental Toxicology University of California Santa Cruz CA USA; ^5^ Complex Carbohydrate Research Center University of Georgia Athens GA USA; ^6^ Department of Pharmaceutical Sciences University of Basel Basel Switzerland

**Keywords:** bacteriophage, mucin glycans, mucus, *Vibrio cholerae*, virulence, Microbiology, Virology & Host Pathogen Interaction

## Abstract

Pandemic and endemic strains of *Vibrio cholerae* arise from toxigenic conversion by the CTXφ bacteriophage, a process by which CTXφ infects nontoxigenic strains of *V. cholerae.* CTXφ encodes the cholera toxin, an enterotoxin responsible for the watery diarrhea associated with cholera infections. Despite the critical role of CTXφ during infections, signals that affect CTXφ‐driven toxigenic conversion or expression of the CTXφ‐encoded cholera toxin remain poorly characterized, particularly in the context of the gut mucosa. Here, we identify mucin polymers as potent regulators of CTXφ‐driven pathogenicity in *V. cholerae.* Our results indicate that mucin‐associated *O‐*glycans block toxigenic conversion by CTXφ and suppress the expression of CTXφ‐related virulence factors, including the toxin co‐regulated pilus and cholera toxin, by interfering with the TcpP/ToxR/ToxT virulence pathway. By synthesizing individual mucin glycan structures *de novo*, we identify the Core 2 motif as the critical structure governing this virulence attenuation. Overall, our results highlight a novel mechanism by which mucins and their associated *O*‐glycan structures affect CTXφ‐mediated evolution and pathogenicity of *V. cholerae*, underscoring the potential regulatory power housed within mucus*.*

## Graphical abstract

Mucins suppress *V. cholerae* virulence in a manner dependent on their core 2 glycan structure.

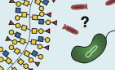

## Introduction

Bacteriophages play a critical role in shaping the virulence and evolution of bacterial pathogens. One striking example of this phenomenon can be seen with *Vibrio cholerae*, the causative agent of cholera, an acute dehydrating diarrheal disease that infects millions of people each year. While this microbe naturally inhabits water bodies such as rivers and ponds (Reidl & Klose, 2002), *V. cholerae* can transform into a virulent enteric pathogen through a process known as toxigenic conversion, in which the CTXφ bacteriophage infects nontoxigenic strains of *V. cholerae* (Waldor & Mekalanos, 1996). CTXφ encodes both subunits of the cholera toxin, a potent enterotoxin that triggers the massive and watery diarrhea that is the hallmark of cholera infections (Waldor & Mekalanos, 1996). While toxigenic conversion is thought to occur in both natural aquatic habitats and within the gastrointestinal tracts of hosts during infection (Waldor & Mekalanos, 1996; Faruque *et al*, 1999; Faruque & Nair, 2002; Reidl & Klose, 2002), the signals that affect CTXφ toxigenic conversion or the expression of the CTXφ‐encoded cholera toxin have remained largely unclear.

Mucus is a complex ecological niche colonized by *V. cholerae* during infection. *V. cholerae* encounters a multitude of host‐derived signals during infection of the gut mucosa, including oxygen limitation (Liu *et al*, 2011), bile salts (Hung & Mekalanos, 2005), and small molecules produced by the host microbiota (Qin *et al*, 2020). While each of these signals impacts the virulence program of *V. cholerae*, one set of molecules in the host mucosa that is often overlooked in studies of host–pathogen interactions is that of mucin glycoproteins, the major gel‐forming units of mucus. These abundant molecules, which reach millimolar concentrations in mucus, play a multi‐faceted role in the gut, providing nutrients and attachment sites for the microbiota while simultaneously protecting the body from invading enteric pathogens (Wagner *et al*, 2018). Recent work in an infant mouse model of infection has shown that *V. cholerae* preferentially colonizes regions of the gut that produce lower amounts of mucins (Millet *et al*, 2014). It has also been reported that the destruction of mucins by the mucolytic agent *N*‐acetyl‐l‐cysteine in infant mice leads to a dramatic increase in *V. cholerae* burden in the small intestines (Millet *et al*, 2014). Together, these observations strongly suggest that mucins typically play a critical role in protecting the gut from *V. cholerae* infections. However, the molecular mechanisms by which mucins exert these protective effects have not yet been fully explored.

Here, we utilize biochemical fractionation of native mucus samples to show that mucins and their associated glycans inhibit toxigenic conversion by the CTXφ bacteriophage by downregulating expression of the toxin co‐regulated pilus [TCP], which serves as a CTXφ receptor. Our results further indicate that mucin glycans directly suppress expression of the CTXφ‐encoded cholera toxin by interfering with the ToxR/TcpP/ToxT regulatory pathway; we support these results with enzyme‐linked immunosorbent assay [ELISA]‐based protein assays and *in vitro* infection assays of human epithelial cells. We also synthesize individual mucin glycan structures *de novo* and find that the Core 2 glycan motif, which is abundant in mucus, is sufficient for this virulence attenuation. Overall, our results shed light on a novel mechanism by which mucins and their associated glycans may hinder CTXφ‐mediated evolution and pathogenicity of toxigenic *V. cholerae*.

## Results

### Mucins and their associated glycans inhibit toxigenic conversion by the CTXφ bacteriophage

**Figure 1:**
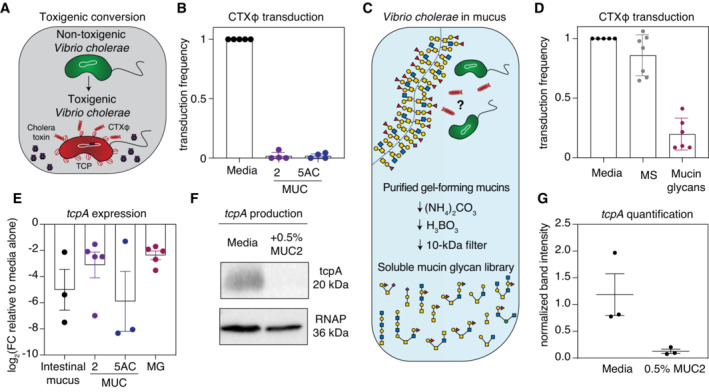
Mucins and their associated glycans inhibit toxigenic conversion and downregulate the TCP Schematic of *V. cholerae* toxigenic conversion by CTXφ. Cholera toxin shown in purple; CTXφ indicated as red rods; nontoxigenic *V. cholerae* shown in green; *V. cholerae* after acquisition of cholera toxin genes shown in red.Mucins reduce CTXφ‐Km transduction efficiency. Bars indicate mean ± standard error of the mean [SEM], with individual measurements of biological replicates shown.Mucins were purified from intestinal and gastric mucus. Complex mucin glycans were isolated from mucin polymer gels using alkaline β‐elimination (Materials and Methods), leaving their reducing ends intact. *V. cholerae* and CTXφ phage are labeled as in (A).A pool of purified mucin glycans suppresses CTXφ‐Km transduction. Bars indicate mean ± SEM, with individual measurements of biological replicates shown.Whole intestinal mucus, natively purified mucins (MUC2 and MUC5AC), and a pool of isolated mucin glycans (MG) downregulate expression of the TCP‐encoding *tcpA* gene relative to media alone. Gene expression was measured by qRT‐PCR and normalized to a control gene (*gyrA*). Bars indicate mean ± SEM, with individual measurements of biological replicates shown. FC, fold change.TcpA production in *V. cholerae* measured by Western blot of cells grown in the presence or absence of mucins. Cell pellets (normalized by total protein amounts) were subjected to Western blot analysis with anti‐TcpA antibody.Quantification of tcpA Western blots from *n* = 3 biological replicates. Bars indicate mean ± SEM, with individual measurements shown. Source data are available online for this figure.

Although toxigenic conversion by CTXφ transforms *V. cholerae* into a hyper‐virulent pathogen (Fig 1A), the signals that influence this process have not been well documented, particularly in the context of the host mucosa. Given the abundance of mucin glycoproteins in mucus, as well as the protective effects of mucins that have been recently observed in an infant mouse model of cholera (Millet *et al*, 2014), we hypothesized that mucins interfere with CTXφ‐mediated toxigenic conversion. To test this hypothesis, we first natively purified MUC5AC and MUC2 mucins from porcine gastrointestinal tracts. Porcine mucus represents a commonly used model system for studying the mucosal niche in higher vertebrates due to strong similarities between human‐ and porcine‐derived mucin structure and glycosylation (Turner *et al*, 1999; Celli *et al*, 2005; Jin *et al*, 2017). CTXφ encodes repressors of CTXφ replication, conferring protection from secondary infection (Kimsey & Waldor, 1998; Kim *et al*, 2017). Thus, to measure toxigenic conversion by CTXφ, we quantified the ability of CTXφ harboring a kanamycin‐resistance cassette to infect a susceptible *V. cholerae* strain lacking the CTXφ repressor (Gallego‐Hernandez *et al*, 2020), in the presence or absence of purified mucins. Strikingly, a physiologically relevant concentration of 0.5% w/v (Bansil & Turner, 2018) purified MUC5AC or MUC2 ablated the ability of CTXφ to infect the susceptible *V. cholerae* strain compared with control media (Fig 1B), suggesting that mucins strongly block toxigenic conversion by CTXφ.

We next sought to identify the molecular motifs in mucins that suppress CTXφ‐mediated toxigenic conversion. Mucin glycans, which comprise 50–90% of the molecular mass of mucins, are promising molecules for regulating host–microbe interactions (Robbe *et al*, 2004; Jin *et al*, 2017). We chemically isolated mucin glycans via nonreductive, alkaline β‐elimination, yielding a library of structurally intact glycans released from the MUC5AC polymer (Fig 1C). To determine whether mucin glycans also suppress toxigenic conversion, we repeated the CTXφ transduction assay in the presence or absence of 0.1% w/v purified MUC5AC glycans and found that this physiologically relevant concentration of mucin glycans was sufficient to decrease CTXφ transduction (Fig 1D). In contrast, we found that a pool of 0.1% w/v mucin monosaccharides did not inhibit phage transduction, strongly suggesting that the complex structure of mucin glycans is critical for this phenotype (Fig 1D).

**Figure EV1:**
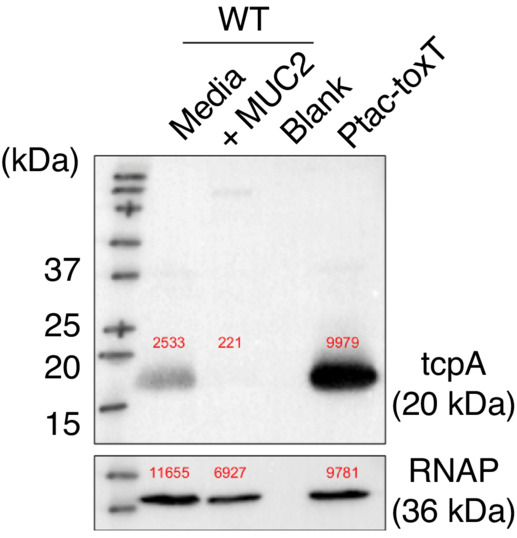
Representative western blot image of TcpA from *V. cholerae* grown in the presence or absence of mucins, or in the IPTG‐inducible *toxT* strain TcpA production in *V. cholerae* measured by Western blot of cells grown in the presence or absence of mucins. Cell pellets (normalized by total protein amounts) were subjected to Western blot analysis with anti‐TcpA antibody. Band intensities are shown above each band. The image is derived from the same experiment as in Fig 1F.Source data are available online for this figure.

Our next goal was to elucidate the mechanisms by which mucins and their associated glycans block CTXφ transduction. CTXφ uses the TCP as its receptor, and previous work has shown that toxigenic conversion is largely ablated in strains that lack the TCP (Waldor & Mekalanos, 1996). Thus, we hypothesized that mucin glycans block CTXφ phage transduction by downregulating TCP expression. To test this hypothesis, we incubated *V. cholerae* with whole intestinal porcine mucus, 0.5% w/v purified MUC5AC or MUC2 mucin, or a pool of isolated 0.1% w/v mucin glycans and then used quantitative real‐time polymerase chain reaction [qRT‐PCR] to measure expression levels of the pilus‐encoding *tcpA* gene. Each sample downregulated *tcpA* expression by ~ 5‐ to 30‐fold on average relative to a medium‐only control (Fig 1E). To further confirm these gene expression results, we used a Western blot to directly measure the levels of TcpA protein in the presence or absence of 0.5% w/v purified mucin and found that MUC2 decreased TcpA protein levels by ~ 10 fold on average (Figs 1F and G and EV1). Together, these results suggest that downregulation of the TCP phage receptor is one mechanism by which mucins block toxigenic conversion by the CTXφ.

### Mucin *O*‐glycans downregulate the expression and production of cholera toxin

**Figure 2:**
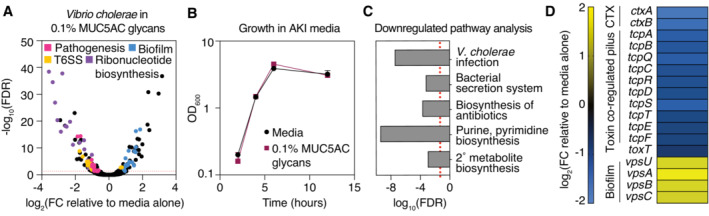
Mucin glycans trigger global changes in the transcriptome of *V. cholerae* MUC5AC glycans elicit a global transcriptional response in *V. cholerae*. A complete list of fold change [FC] values and false discovery rate [FDR]‐adjusted *P*‐values is provided in Dataset EV1. FC data are average measurements from *n* = 2 biologically independent replicates. FDR‐adjusted *P*‐values were determined using the Benjamini–Hochberg *P*‐value adjustment method. The dotted line represents the threshold for significance (FDR‐adjusted *P* < 0.05).Growth is not altered by the presence of mucin glycans in AKI medium. Data are the mean OD value at 600 nm (OD_600_) ± standard error of the mean for *n =* 3 biologically independent replicates.Functional enrichment analyses reveal key virulence pathways among downregulated genes. Significance of enrichment was calculated from the Mann–Whitney *U*‐test followed by the Benjamini–Hochberg procedure for multiple corrections for mean log_2_‐transformed FCs from *n* = 2 biologically independent replicates. The dotted line represents the threshold for significance (FDR‐adjusted *P* < 0.05).RNA‐seq data for selected genes belonging to *V. cholerae* CTXφ‐mediated pathogenesis and biofilm formation. A complete list of FC values and FDR‐adjusted *P*‐values is provided in Dataset EV1. FC data are average measurements from *n* = 2 biologically independent replicates.

Given the significant downregulation of *tcpA* by mucins and mucin glycans, we reasoned that mucin glycans may also trigger differential expression of other *V. cholerae* genes. To determine whether mucin glycans induce changes in gene expression beyond the TCP, we performed RNA sequencing [RNA‐seq] of *V. cholerae* in AKI medium with or without 0.1% w/v MUC5AC glycans and assessed the extent to which glycans alter the global transcriptome of *V. cholerae*. A pooled library of MUC5AC glycans triggered global changes in gene expression, significantly upregulating 57 genes and downregulating 95 genes (Fig 2A) compared with cells grown in medium alone (Dataset EV1), without altering growth (Fig 2B). Pathways associated with pathogenesis, ribonucleotide (purine and pyrimidine) metabolism, and an auxiliary type VI secretion system [T6SS] exhibited downregulation while pathways encoding biofilm‐related genes showed upregulation (Fig 2C). In agreement with our previous qRT‐PCR experiment (Fig 1E), our RNA‐seq dataset showed that TCP‐encoding genes were significantly downregulated (Fig 2D).

**Figure 3:**
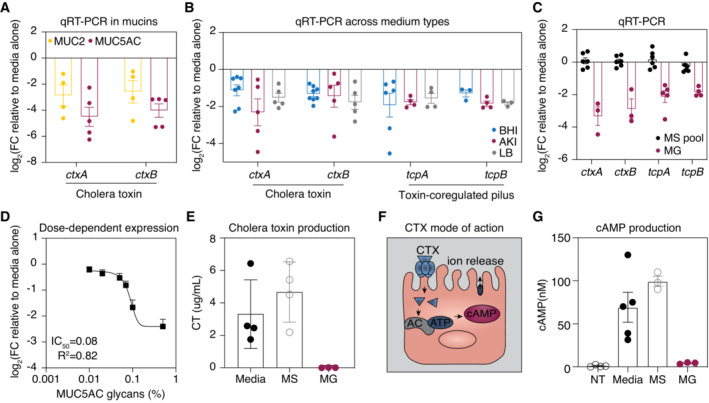
Mucin glycans downregulate the expression of CTXφ‐associated virulence genes and suppress cholera toxin production Full‐length mucins downregulate the expression of cholera toxin relative to media alone. Gene expression was measured by qRT‐PCR and normalized to a control gene (*gyrA*). Bars indicate mean ± standard error of the mean [SEM], with individual measurements of biological replicates shown. FC, fold change.Mucin glycans downregulate the expression of cholera toxin and TCP‐encoding genes in three different medium conditions. Gene expression was measured by qRT‐PCR and normalized to a control gene (*gyrA*). Bars indicate mean ± SEM, with individual measurements of biological replicates shown. BHI, brain heart infusion broth.Complex mucin glycans (MG), but not their monosaccharide components (MS pool), induce expression changes in CTXφ‐associated virulence genes relative to media (AKI) alone. Gene expression was measured by qRT‐PCR and normalized to a control gene (*gyrA*). Bars indicate mean ± SEM, with individual measurements of biological replicates shown.Mucin glycans regulate *ctxA* expression in a dose‐dependent manner. Gene expression was measured by qRT‐PCR and normalized to a control gene (*gyrA*). Points indicate mean ± SEM, with the average of *n* = 3 biological replicates shown. A nonlinear antagonist binding best‐fit curve gives a half‐maximum inhibitory concentration (IC_50_) of 0.087 wt% (*R* ^2^ = 0.82).Complex mucin glycans, but not their monosaccharide (MS) components, suppress cholera toxin production. Bars indicate mean ± SEM, with individual measurements of biological replicates shown.Schematic of the cholera toxin mode of action. Cholera toxin triggers adenylate cyclase (AC) activity in human epithelial cells leading to elevated cAMP levels and ATP‐mediated efflux of ions and water from enterocytes.Supernatant taken from *V. cholerae* grown in the presence of mucin glycans (MG) induces lower levels of cAMP production in HT‐29 cells compared with supernatant taken from *V. cholerae* grown in the presence of monosaccharides (MS) or media alone. Bars indicate mean ± SEM, with individual measurements of biological replicates shown. NT, no treatment.

Importantly, transcriptome analysis indicated that mucin glycans significantly downregulated genes encoding both subunits of the cholera toxin, *ctxA* and *ctxB* (Fig 2D). Consistent with this observation, cholera toxin‐encoding genes also exhibited downregulation in the presence of full‐length MUC5AC or MUC2 mucins, as measured by qRT‐PCR (Fig 3A). We also confirmed that mucin glycans suppress the expression of both the TCP and the cholera toxin across three different medium types (Fig 3B). Importantly, the addition of a pool of monosaccharides found in mucins did not alter the expression of virulence genes (Fig 3C), suggesting that the complex structure of mucin glycans is critical to their function. Furthermore, we found that mucin glycans downregulated *ctxA* expression in a dose‐dependent manner (Fig 3D), with suppression occurring at concentrations below those found in mucosal surfaces (Bansil & Turner, 2018). To confirm that mucin glycans suppress cholera toxin production, we used ELISA‐based assays to directly measure the levels of cholera toxin protein produced in the supernatant of *V. cholerae* grown in the presence or absence of mucin glycans. Strikingly, exposure to mucin glycans resulted in a nearly 100‐fold reduction of cholera toxin production relative to a medium‐only control, while equivalent concentrations of monosaccharide had no effect (Fig 3E). Together, these results suggest that the complex glycan structures found in the mucus barrier are sufficient for the downregulation of CTXφ‐related virulence genes.

### Mucin glycans suppress cAMP production in human epithelial cells

Cholera toxin binds and enters intestinal epithelial cells to activate its target, adenylate cyclase, which then elevates cyclic adenosine monophosphate [cAMP] levels within host cells (Fig 3F). This action then triggers a rapid efflux of chloride and a decreased uptake of sodium ions, leading to massive water secretion through the intestinal cells and causing severe diarrhea, which is the hallmark of cholera infections (De Haan & Hirst, 2004). We hypothesized that the decreased toxin production observed in *V. cholerae* grown in the presence of mucin glycans should also reduce cAMP levels in epithelial cells. To test this hypothesis, we grew *V. cholerae* in the presence or absence of mucin glycans, incubated the supernatant of these samples with HT‐29 human epithelial cells for 4 h, and then analyzed cAMP levels by ELISA. As a control, we first confirmed that HT‐29 cells did not produce cAMP when exposed to a medium‐only control not treated with *V. cholerae*. In contrast, the addition of *V. cholerae* supernatant from bacteria grown in the absence of mucin glycans strongly stimulated cAMP production in HT‐29 cells (Fig 3G). Strikingly, supernatant taken from *V. cholerae* grown in the presence of mucin glycans triggered significantly lower levels of cAMP production compared with supernatant taken from *V. cholerae* grown in the absence of mucin glycans, while monosaccharides had no effect relative to this same control (Fig 3G). Together, these results confirm that mucin glycans suppress CTXφ‐mediated virulence of *V. cholerae* by inhibiting cholera toxin‐driven cAMP production in human epithelial cells.

### Mucin glycans interfere with the TcpP/ToxR/ToxT pathway to suppress virulence gene expression

**Figure 4:**
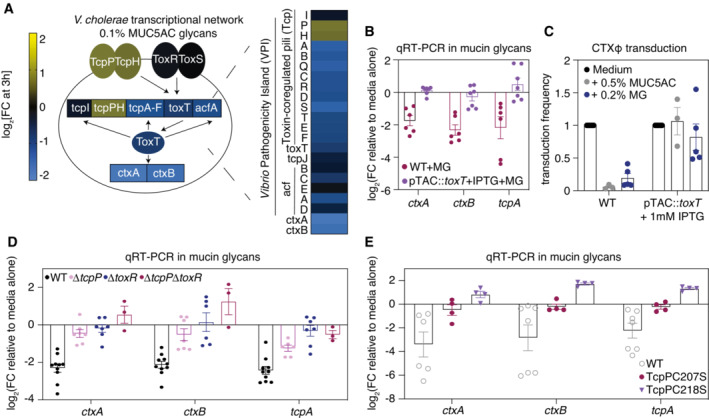
Mucin glycans downregulate the expression of CTXφ‐associated virulence genes by interfering with the TcpP/ToxR/ToxT pathway ASchematic overview of the regulation of cholera toxin (*ctxAB*) expression in *V. cholerae*, with each component colored according to its relative fold change [FC] in the presence of mucin glycans. Genes on the *Vibrio* pathogenicity island are specifically highlighted. A complete list of FC values and false discovery rate [FDR]‐adjusted *P*‐values is provided in Dataset EV1. FC data are average measurements from *n* = 2 biologically independent replicates.BMucin glycans (MG) are unable to downregulate the expression of CTXφ‐associated virulence genes when *toxT* is constitutively expressed in a strain in which the native promoter for *toxT* has been replaced with an IPTG‐inducible promoter. Gene expression was measured by qRT‐PCR and normalized to a control gene (*gyrA*). Bars indicate mean ± standard error of the mean [SEM], with individual measurements of biological replicates shown. WT, wild type.CMucins and mucin glycans reduce CTXφ‐Km transduction efficiency in a toxT‐dependent manner. Bars indicate mean ± SEM, with individual measurements of biological replicates shown.D, EMucin glycans (MG) are unable to downregulate the expression of CTXφ‐associated virulence genes in mutants that modulate *toxT* expression. Gene expression was measured by qRT‐PCR and normalized to a control gene (*gyrA*). Bars indicate mean ± SEM, with individual measurements of biological replicates (*n* = 3–9) shown. WT, wild type.

**Figure EV2:**
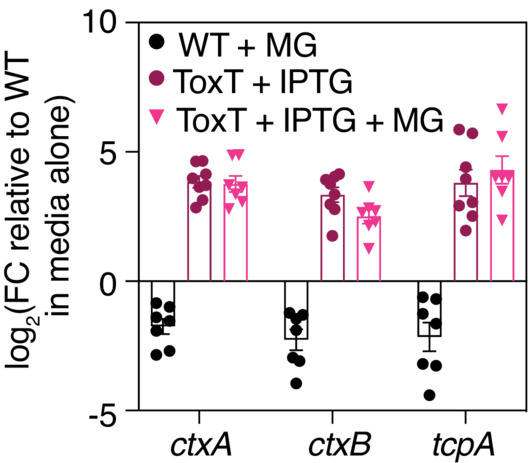
Measurement of CTX‐associated virulence gene expression in the IPTG‐inducible *toxT* strain Induction of toxT with 1 mM of IPTG increases the expression of virulence genes relative to the wild‐type [WT] strain. Gene expression was measured by qRT‐PCR and normalized to a control gene (*gyrA*). Bars indicate mean ± standard error of the mean, with individual measurements of biological replicates (*n* = 6–8) shown. MG, mucin glycans.

We sought to further clarify the mechanisms by which mucin glycans downregulate cholera toxin and TCP. Both of these virulence factors act downstream of ToxT, a central regulator of CTXφ‐mediated pathogenicity in *V. cholerae* (Kumar *et al*, 2020). Our RNA‐seq results indicate that mucin glycans downregulate *toxT* expression to a modest but statistically significant extent (~ 1.5 fold change, *P* = 0.03; Fig 4A).To clarify the role of ToxT in mucin glycan‐dependent downregulation of CTXφ‐related virulence genes, we incubated mucin glycans with a *V. cholerae* mutant in which the native promoter for *toxT* was replaced with an isopropyl β‐d‐1‐thiogalactopyranoside [IPTG]‐inducible promoter, which allows for its constitutive expression in the presence of IPTG (Gallego‐Hernandez *et al*, 2020). Using this strain, we found that the ability of mucin glycans to downregulate the cholera toxin and the TCP was completely ablated when *toxT* expression was constitutively induced by 1 mM IPTG (Fig 4B), strongly indicating that the virulence‐suppressing effects of mucin glycans are dependent on their ability to suppress *toxT* gene expression. To further probe the role of ToxT, we repeated the CTXφ transduction assays with the IPTG‐inducible *toxT‐*expressing strain. Constitutive activation of toxT leads to high levels of TcpA production (Figs EV1 and EV2), which is the phage receptor for CTXφ. When *toxT* was constitutively expressed, we found that mucin glycans were no longer able to block CTXφ phage transduction despite high levels of TcpA production (Fig 4C), indicating that the primary mechanism underlying the glycan response is likely due to changes in *toxT* gene expression rather than steric hindrance or glycan‐mediated binding to TcpA.

Research has shown that two membrane‐localized complexes (TcpP/H and ToxR/S) promote the transcription of *toxT* (Fig 4A; Yoon & Waters, 2019). Our RNA‐seq results indicate that mucin glycans do not suppress gene expression of *tcpP*/*H* or *toxR*/*S*, which raises the possibility that mucin glycans downregulate *toxT* gene expression by interfering with the activity of these upstream regulators. To test this hypothesis, we incubated mucin glycans with a variety of mutants defective in the regulation of *toxT* gene expression, including single‐deletion mutants (Δ*tcpP*, Δ*toxR*), a double‐deletion mutant (Δ*tcpP*Δ*toxR*), and single‐point mutations that have previously been shown to ablate TcpP signaling (C207S, C218S; Yang *et al*, 2013). Importantly, we found that the ability of mucin glycans to downregulate downstream CTXφ‐related virulence genes was impaired or completely ablated in each of these upstream mutants (Fig 4D and E), suggesting that mucin glycans likely act through these regulatory components to suppress the expression of downstream *tcp* and *ctxAB* genes.

### Characterization of synthetic mucin *O*‐glycans reveals that individual glycans possessing the Core 2 motif are sufficient for virulence attenuation

**Figure 5:**
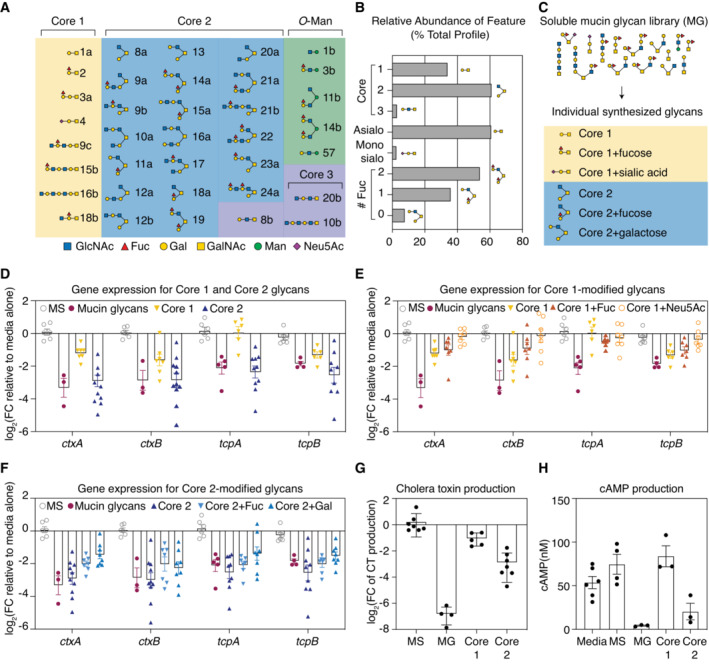
Synthesized Core 2 glycan structures are sufficient to downregulate the expression of CTXφ‐associated virulence genes O‐Glycan structural diversity detected by NSI‐MS analysis (Materials and Methods) of the *O*‐linked glycans released from MUC5AC glycans. Glycan structures depicted are over > 0.1% of the relative abundance of the glycan pool. Fuc, fucose; Gal, galactose; GalNAc, *N*‐acetylgalactosamine; GlcNAc, *N*‐acetylglucosamine; Man, mannose; Neu5Ac, *N*‐acetylneuraminic acid.Relative abundances of core structural features in the MUC5AC glycan pool. The complex mucin glycan pool is dominated by Core 1‐ and Core 2‐derived glycan structures that are heavily fucosylated and sparsely sialylated.Depiction of synthesized mucin glycan structures that are abundant in the complex mucin glycan pool.Exposure to Core 2 decreases the expression of cholera toxin‐ and TCP‐encoding genes, while Core 1 elicits a weaker effect. Gene expression was measured by qRT‐PCR and normalized to a control gene (*gyrA*). Bars indicate mean ± standard error of the mean [SEM], with individual measurements of biological replicates (*n* = 3–11) shown. MS, monosaccharides.Addition of fucose or sialic acid to Core 1 does not strongly alter the expression of cholera toxin‐ and TCP‐encoding genes, compared with Core 1 alone. Gene expression was measured by qRT‐PCR and normalized to a control gene (*gyrA*). Bars indicate mean ± SEM, with individual measurements of biological replicates (*n* = 3–7) shown. MS, monosaccharides.Addition of fucose or galactose to Core 2 does not strongly alter the expression of cholera toxin‐ and TCP‐encoding genes, compared with Core 2 alone. Gene expression was measured by qRT‐PCR and normalized to a control gene (*gyrA*). Bars indicate mean ± SEM, with individual measurements of biological replicates (*n* = 3–10) shown. MS, monosaccharides.Core 2 glycans reduce cholera toxin production by nearly 10‐fold, while Core 1 glycans reduce toxin production by 2‐fold. Cholera toxin production was measured using a GM1‐ELISA‐based assay. Bars indicate mean ± SEM, with individual measurements of biological replicates shown. MG, mucin glycans; MS, monosaccharides.Supernatant taken from *V. cholerae* grown in the presence of Core 2 glycans induces lower levels of cAMP production in HT‐29 cells compared with supernatant taken from *V. cholerae* grown in the presence of Core 1 glycans, a pool of monosaccharides (MS), or media alone. Bars indicate mean ± SEM, with individual measurements of biological replicates shown. MG, mucin glycans; MS, monosaccharides.

We next sought to identify specific glycan motifs responsible for the downregulation of CTXφ‐related virulence genes in *V. cholerae*. Using nanospray ionization multidimensional mass spectrometry (NSI‐MSn, Thermo Fisher Orbitrap Discovery), we characterized > 80 glycan structures in our collection of purified mucin glycans, including isobaric glycans with distinct structural characteristics (glycans with identical mass but different isomeric configurations; Fig 5A). Overall, the MUC5AC glycan pool was dominated by Core 1‐ and Core 2‐type *O*‐glycan structures that were partially modified by fucose (Fig 5B) and sparsely sialylated (Fig 5B). In particular, six glycan structures (Core 1, Core 1 + Fuc, Core 1 + sialic acid, Core 2, Core 2 + Fuc, and Core 2 + Gal) represented > 40% of the total glycan profile and were thus identified as candidates for virulence‐attenuating activity (Fig 5C).

Rather than fractionating glycan pools down to the single‐glycan level, which poses technical challenges (Cummings & Pierce, 2014), we chemically synthesized these six highly abundant mucin glycans *de novo*. We first focused on Core 1 and Core 2, the two most abundant core structures, which are the foundation upon which more complex glycans are built. To determine whether Core 1 and Core 2 have a regulatory capacity similar to that of the complex glycan pool, we incubated each structure with *V. cholerae* and used qRT‐PCR to measure the expression of CTXφ‐related virulence genes. Strikingly, the synthetic Core 2 structure downregulated the expression of these virulence genes to a similar degree as the complex glycan pool, while the Core 1 structure was comparatively less potent (Fig 5D). Moreover, the addition of fucose, galactose, and sialic acid onto these Core 1 (Fig 5E) and Core 2 (Fig 5F) structures did not significantly alter the observed changes in gene expression, suggesting that the underlying Core 2 motif is sufficient for virulence downregulation.

**Figure EV3:**
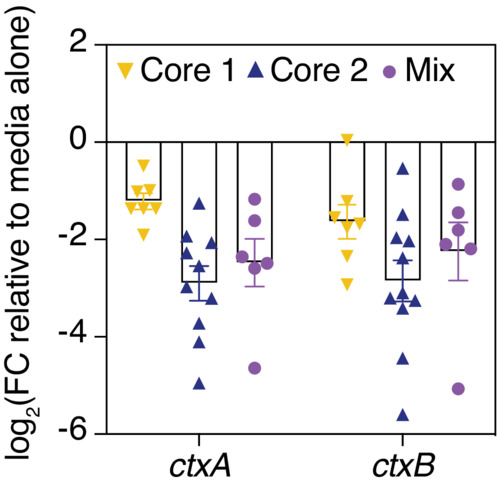
The combination of Core 1 and Core 2 (mix) does not strongly alter the expression of cholera toxin‐ and TCP‐encoding genes, compared with the Core 2 structure alone Gene expression was measured by qRT‐PCR and normalized to a control gene (*gyrA*). Bars indicate mean ± SEM, with individual measurements of biological replicates (*n* = 6–11) shown. FC, fold change.

To further probe the importance of the Core 2 motif, we performed ELISA to measure the levels of secreted cholera toxin in *V. cholerae* cultures incubated in the presence or absence of unmodified Core 1 and Core 2. We found that Core 2 reduced cholera toxin production by nearly 10‐fold relative to a medium control, while Core 1 glycans reduced toxin production by only 2‐fold in this same assay (Fig 5G). We repeated our tissue culture infection assays and found that supernatant taken from *V. cholerae* incubated with the Core 2 structure decreased the levels of cAMP production in HT‐29 cells by 60% relative to supernatant taken from *V. cholerae* grown in medium alone, while there were no significant changes in cAMP production when the Core 1 structure was used in these same experiments (Fig 5H). Finally, we found that a combination of Core 1 and Core 2 structures did not further decrease CTXφ‐related virulence gene expression compared with the Core 2 structure alone (Fig EV3). Together, these results suggest that the downregulation of cholera toxin and the TCP is largely driven by the Core 2 motif.

## Discussion

The pathogenicity of *V. cholerae* can largely be attributed to toxigenic conversion by the CTXφ bacteriophage, which encodes the cholera toxin and uses the TCP as its phage receptor. In this work, by biochemically fractionating whole mucus samples, we demonstrated that natively purified mucins and their associated glycans inhibit toxigenic conversion by the CTXφ phage, likely by suppressing expression of the TCP phage receptor. In addition, our results indicate that mucin glycans downregulate the CTXφ‐encoded cholera toxin by interfering with the TcpP/ToxR/ToxT regulatory pathway. By synthesizing individual mucin glycan structures *de novo*, we determined that the Core 2 glycan structure is specifically responsible for this observed virulence attenuation. Together, our results identify a novel mechanism through which mucins may interfere with CTXφ‐driven pathogenicity of toxigenic *V. cholerae*.

As *V. cholerae* infects the gut mucosa, this pathogen will undoubtably encounter mucins and their associated glycans during intestinal colonization, which are present at millimolar concentrations in the gastrointestinal tract. Interestingly, our RNA‐seq results suggest that *V. cholerae* dramatically rewires its transcriptome in response to mucin glycans, with over 150 genes exhibiting significant changes in gene expression. While our experiments here focused on the CTXφ‐related TCP and cholera toxin genes, we also observed that mucin glycans trigger gene expression changes in other pathways, including biofilm formation, type VI secretion, and ribonucleotide metabolism. Interestingly, previous work has linked an upregulation of biofilm gene expression to increased expression of CTXφ‐related virulence genes (Gallego‐Hernandez *et al*, 2020); however, here we found that mucin glycans lead to increased expression of biofilm genes while simultaneously suppressing the expression of *tcp* and *ctx* genes, suggesting that mucin glycans interfere with this virulence regulation. Future work characterizing the molecular details of these gene expression changes may shed light on how *V. cholerae* alters its behavior in mucus.

A few other recent studies have investigated how mucins impact *V. cholerae* physiology. For example, researchers have reported that mucins activate the T6SS and alter motility in *V. cholerae* (Liu *et al*, 2008, 2015; Bachmann *et al*, 2015; Frederick *et al*, 2020). However, these studies utilized commercially available mucins that are subject to harsh chemical treatments, resulting in mucins that are partially degraded and de‐glycosylated (Lieleg *et al*, 2012; Wagner *et al*, 2018). Some of these previous studies also used commercial mucins collapsed onto two‐dimensional agar surfaces, which does not recapitulate the normal three‐dimensional structure of mucus. In contrast, our work uses natively purified mucins to study *V. cholerae* pathogenicity, in which the native structure and function of these large glycoproteins are preserved. In addition, we observed that whole‐length mucins, isolated mucin glycans, and chemically synthesized Core 2 structures all downregulate the cholera toxin and TCP, which strengthens our conclusions regarding the virulence‐attenuating properties of mucins reported in this work.

Bacteriophages contribute to the virulence of many other bacterial pathogens beyond *V. cholerae*. For example, *Salmonella* Typhimurium harbors prophages that encode type III secretion system effectors (Coombes *et al*, 2005), *Clostridium botulinum* encodes the botulinum neurotoxin on a prophage (Fortier, 2017), and enterohemorrhagic *Escherichia coli* [EHEC] is infected by a Shiga‐toxin‐encoding phage (Węgrzyn & Muniesa, 2021) that triggers the symptoms associated with EHEC infections. Each of these pathogens invades mucosal niches during infection and thus encounters mucins and their associated *O*‐glycans in the human body. Determining whether mucin glycans alter the expression of these other phage‐associated virulence genes will be an important area of future research.

Our results are generally consistent with recent work in murine models of infection that have highlighted the protective roles of mucins *in vivo* (Millet *et al*, 2014). While infant mouse models of cholera infection have certain limitations, including the absence of severe diarrhea and an underdevelopment of host‐defense systems (Sawasvirojwong *et al*, 2013), it has been observed that over 50% of cholera infections in humans are asymptomatic, suggesting that mucus may also actively suppress the CTXφ‐mediated pathogenicity of *V. cholerae* during humans infections (Baker‐Austin *et al*, 2018). Intriguingly, this observation also suggests that ~ 50% of humans infected with *V. cholerae* still develop diarrhea in the presence of mucins. The molecular details that underlie symptomatic vs. asymptomatic infections represent a fascinating area of future study and could reflect differences in the mucus environment. As mucin glycans suppress the virulence of *V. cholerae* without affecting its viability, these glycans may serve as host‐derived anti‐virulence candidates for treating cholera infections that do not select for drug resistance. Overall, our results highlight the potential role that mucins play in altering CTXφ‐mediated evolution and pathogenicity of *V. cholerae* and underscore the wealth of biochemical information and regulatory power that is housed within mucus.

## Materials and Methods

### Bacterial strains and conditions

We grew *V. cholerae* strains overnight in Luria broth [LB] at 37°C under shaking conditions. For all assays, strains were diluted 1:50 into AKI media (Iwanaga *et al*, 1986) in a 96‐well plate (50 μl per well) and statically incubated in the presence or absence of mucus‐derived signals for 3 h under anaerobic conditions, unless otherwise indicated. For all experiments using purified mucins and/or mucin glycans, we used a concentration of 0.5% w/v mucin or 0.1% w/v mucin glycans, which reflects the physiological concentration of mucins and glycans in the gastrointestinal tract (Bansil & Turner, 2006), unless otherwise indicated. The strains and primers used are listed in Tables EV1 and EV2.

### Gene deletion and promoter replacement

To generate deletion constructs, we assembled two DNA fragments of approximately 500 base pairs [bp], including upstream and downstream sequences containing the truncated gene, into the suicide plasmid pGP704sac28. For the *toxT* promoter replacement construct, approximately 500 bp from the upstream gene of *toxT*, the Ptac promoter from pMMB67EH, and the whole *toxT* ORF sequence were assembled together and cloned into pGP704sacB. Plasmids were mobilized into *V. cholerae* A1552 by biparental mating, and a genetic knock‐out/knock‐in procedure was performed as previously described (Lim *et al*, 2006). Briefly, overnight cultures of the donor and recipient strains were mixed 1:1, and mating spots were grown on LB agar plates (37°C, 12 h). We selected transconjugants on LB agar plates containing rifampicin (100 μg/ml) and ampicillin (100 μg/ml). Cointegrants were streak‐purified under selective antibiotics and then grown in LB media without antibiotics. The cultures were streaked onto LB agar plates without NaCl, containing sucrose at a final concentration of 10% (w/v), and incubated for 2 days at room temperature. We determined loss of the wild‐type gene or replacement of the *toxT* promoter region by colony PCR.

### Western blot analyses

We pelleted cells from each condition at 5,000 *g* for 10 min. After the supernatant was decanted, the pellets were resuspended in 60 μl of B‐PER (Thermo Scientific) containing complete Mini ethylenediaminetetraacetic‐acid‐free protease inhibitor cocktail (Roche), and cells were lysed for 30 min at room temperature. After pelleting the cell debris by centrifugation at 15,000 *g* for 5 min, we transferred 40 μl of clear lysate to a new tube. We determined protein concentrations using a Bradford assay reagent (Thermo Scientific) and denatured proteins by adding 10% sodium dodecyl sulfate [SDS] (Sigma‐Aldrich) to a final concentration of 2%. Next, 60 μg of total protein from each cell lysate were loaded and separated by 12% SDS‐polyacrylamide gel electrophoresis. The proteins were transferred onto a polyvinylidene fluoride membrane (Immobilon, 0.45 μm, Millipore) and probed via immunoblotting. We used the following antibodies for immunoblotting: rabbit anti‐TcpA (Taylor *et al*, 2004; 1:2,500), mouse anti‐RnaP (Biolegend; 1:2,500), anti‐rabbit horseradish peroxidase‐conjugated (Promega, 1:2,500), and anti‐mouse horseradish peroxidase‐conjugated (Invitrogen; 1:2,500). The immunoblots were developed with the SuperSignal West Pico chemiluminescent kit (Pierce).

### Mucin purification

Mucus was scraped from fresh pig stomachs (for MUC5AC) and fresh pig intestines (for MUC2) and solubilized in sodium chloride buffer containing protease inhibitors. Insoluble material was removed via low speed centrifugation at 8,000 *g* for 30 min followed by ultracentrifugation at a relative centrifugal force of 190,000 *g* for 1 h at 4°C (40,000 rpm, Beckman 50.2 Ti rotor with polycarbonate bottles). Mucus was then clarified and desalted with disposable PD‐10 desalting columns (GE). We isolated mucins using size‐exclusion chromatography on a Sepharose CL‐2B column. Mucin fractions were identified based on absorbance at 280 nm and then desalted and concentrated with an Amicon stirred cell pressure‐based concentrator (Sigma) with an Omega ultrafiltration 100‐kDa membrane disc filter (Pall). We then lyophilized the purified mucins for storage at −80°C. Lyophilized mucins were reconstituted by gentle shaking at 4°C overnight in the desired medium.

### Mucin glycan isolation

This study applied nonreductive alkaline β‐elimination ammonolysis to dissociate nonreduced glycans from MUC5AC. We added mucins to 1× phosphate buffered saline [PBS] at a concentration of 30 mg/ml and removed insoluble material by low‐speed centrifugation at 8,000 *g* (7,000 rpm, Sorvall GS‐3 rotor) for 20 min at 4°C. Mucins were then precipitated with 60% (v/v) ethanol, collected by centrifugation, and dissolved in water. Next, dissolved mucin was desalted and concentrated with an Amicon stirred cell pressure‐based concentrator (Sigma) with a 100‐kDa membrane filter disc (Pall) and lyophilized. Lyophilized mucins were dissolved in ammonium hydroxide saturated with ammonium carbonate and incubated at 60°C for 48 h to release oligosaccharide glycosylamines and partially deglycosylated mucins. We removed volatile salts via repeated centrifugal evaporation and separated the oligosaccharide glycosylamines from residual deglycosylated mucins via centrifugal filtration through 10‐kDa molecular weight cut‐off membranes according to the manufacturer's instructions (Amicon Ultracel). The resulting oligosaccharide glycosylamines were converted to reducing oligosaccharide hemiacetals via treatment with boric acid. We removed residual boric acid via repeated centrifugal evaporation from methanol.

### Structural analysis of mucin O‐glycans

Glycans released from MUC5AC were permethylated and analyzed by nanospray ionization tandem mass spectrometry [NSI‐MS/MS] following direct infusion into a linear/orbital hybrid ion trap instrument (Orbitrap‐LTQ Discovery, Thermo Fisher) operated in positive ion mode for nonsulfated glycans or in negative mode for the detection of sulfated glycans, as previously described (Takagi *et al*, 2022). Briefly, we dissolved permethylated *O*‐glycans in 1 mM sodium hydroxide in methanol and water (1:1) for direct infusion. For fragmentation by collision‐induced dissociation in NSI‐MS/MS and NSI‐MSn, a normalized collision energy of 35–40% was applied. We performed detection and relative quantification of the prevalence of individual glycans using the total ion mapping functionality of the Xcalibur software package version 2.0 (Thermo Fisher), as previously described (Aoki *et al*, 2007). Structural representations of mucin glycans were based on topologic features detected following collision‐induced dissociation fragmentation and knowledge of *O*‐glycan biosynthetic pathways. Approximately 40% of the *m*/*z* values reported here were associated with two or three isomeric glycan structures. We utilized NSI‐MS/MS and MSn as needed to assign isomeric heterogeneity at each *m*/*z* value. Graphic representations of glycan monosaccharide residues are consistent with the Symbol Nomenclature For Glycans, as adopted by the glycomics and glycobiology communities (Neelamegham *et al*, 2019). Glycomics data and metadata were obtained and are presented in accordance with MIRAGE standards and the Athens Guidelines (Liu *et al*, 2017). We deposited all raw mass spectrometry data related to mucin glycan profiles at GlycoPost, ID GPST000254 (Watanabe *et al*, 2021).

### Preparation and analysis of synthetic glycans

The Core 1 + sialic acid glycan was prepared according to our synthetic protocols previously described in Takagi *et al* (2022). All other glycans (Core 1, Core 1 + fucose, Core 2, Core 2 + fucose, and Core 2 + galactose) were prepared according to further optimized protocols from Minzer & Hevey (2022). All commercial reagents were used as supplied unless otherwise stated, and anhydrous solvents were either commercially acquired or prepared using standard techniques. Organic solutions were concentrated and/or evaporated to dryness under vacuum in a water bath (< 50°C). Molecular sieves were dried at 400°C under vacuum for 20–30 min prior to use. Amberlite IR‐120H resin was washed extensively with MeOH and dried under vacuum prior to use. Purification of compounds was performed through medium‐pressure liquid chromatography using a CombiFlash Companion (Teledyne ISCO) equipped with either RediSep normal‐phase flash columns (Teledyne ISCO) or self‐packed reversed‐phase C18 columns (LiChroprep RP‐18 resin, 25–40 μm). High‐pressure liquid chromatography [HPLC] analysis was used to assess final purity and was performed using an Agilent 1100 LC equipped with an Atlantis T3 (3 mm, 2.1 × 100 mm) C18 column and an evaporative light scattering detector. HPLC traces of the individual glycan batches used for experiments are available in the Appendix Fig S1.

### Transduction assays

We isolated CTXφ‐kanamycin (Km) by growing *V. cholerae* O395 (pCTXφ‐Km) overnight in 5 ml LB with kanamycin (50 μg/ml), pH 8.5 at 37°C and filtering supernatant fluid through 0.22‐μm filters (Millipore). The CTXφ ΔVC1464 repressor was deleted to eliminate phage immunity. We grew ΔVC1464 anaerobically at 37°C under planktonic conditions. Harvested cells were normalized to an optical density (OD_600_) of 1.0 and mixed with CTXφ‐Km cells for 1 h anaerobically at 37°C. After 1 h, cells were plated on control LB plates and LB plates containing Km (50 μg/ml). Three biological replicates were analyzed for mucin glycans, and two biological replicates were used for mucins with three technical replicates.

### RNA extraction for qRT‐PCR and RNA‐seq experiments

We pelleted 50 μl of cells grown with or without mucin glycans at 12,000 *g* in a tabletop centrifuge for 2 min. Cells were generally grown in AKI media under anaerobic conditions unless otherwise indicated. We resuspended cell pellets in 300 μl of 2× Tissue and Cell Lysis Buffer (Lucigen) with 2 μl of proteinase K. Samples were incubated at 65°C to aid in cell lysis. Next, 175 μl of MPC Protein Precipitation Reagent (Lucigen) were added to the samples, which were spun for 10 min at the maximum speed in a tabletop centrifuge to precipitate out proteins. The supernatant was then mixed with 500 μl of isopropanol and spun down for 15 min at the maximum speed. The resulting pellet of RNA was washed twice with 70% ethanol and centrifuged for 15 min at the maximum speed following each wash. The RNA was then air‐dried for 5 min and dissolved in 50 μl of nuclease‐free water. To remove contaminating DNA from the RNA preparation, we incubated each sample with 5 μl of Ambion RNA Turbo Buffer and 2 μl of Turbo DNAse (Thermo Fisher) for 30 min at 37°C. Next, we added 5 μl of Ambion reaction inactivator to each sample to remove DNAse. Samples were spun at the maximum speed for 2 min, and then supernatant containing purified RNA was collected and stored at −20°C until use.

### qRT‐PCR protocols

To prepare complementary DNA [cDNA] for qRT‐PCR samples, we combined approximately 1 µg of purified RNA with 1 μl of random hexamers (New England Biolabs), 10 μl of ProtoScript II reaction mix (New England Biolabs), and 2 μl of ProtoScript enzyme mix (New England Biolabs). We incubated the samples at 25°C for 5 min and then at 42°C for 1 h to generate cDNA.

The Sybr Fast qPCR 2× Master Mix (Roche) was used to perform qRT‐PCR experiments. Briefly, we incubated approximately 10 ng of cDNA with 5 μl of 2× Sybr (Roche), a final concentration of 300 nM of each primer, and nuclease‐free water. qRT‐PCR experiments were conducted in a 384‐well plate format on a LightCycler 480 system (Roche). We applied a thermocycling program of 95°C for 10 min, 95°C for 15 s, 60°C for 30 s, and 72°C for 30 s, with 40 cycles of steps 2–4. Fold changes were then calculated using the ddCT method relative to the housekeeping gene *gyrA*.

### RNA‐seq protocols

We depleted the purified RNA of ribosomal RNA using the Illumina RiboZero depletion kit. Depleted RNA was submitted to the MIT BioMicro Center for strand‐specific library preparation. We ran the libraries on a HiSeq2000 with paired‐end reads and a read length of 40 nucleotides. BWA was applied to map the reads to the *V. cholerae* type strain, using unique mapping reads. Any read that overlapped with any region of a gene was counted as a read. We then employed DEseq analysis to identify significant changes in gene expression between cells grown in the presence and absence of mucin glycans.

### Cholera toxin ELISA

GM1 ganglioside enzyme‐linked immunosorbent CT assays were performed as previously described (Laboratory Testing for Cholera, https://www.cdc.gov/cholera/laboratory.html) on supernatants from *V. cholerae* grown in medium, mucin glycans, and synthetic glycans. Briefly, we coated 96‐well polystyrene microtiter plates with GM1 ganglioside overnight. We washed the plates three times with PBS (pH 7.4), 0.2% bovine serum albumin [BSA], and 0.05% Tween 20 and then applied 1% (w/v) BSA to block the GM1‐coated plates for 30 min at room temperature. The plates were then washed three times to remove BSA. Next, we added supernatant to the wells and incubated the samples for 1 h at 37°C. Then, 1% BSA was used to block the wells for 30 min at room temperature, and the wells were washed three times thereafter. Subsequently, a goat anti‐CT polyclonal antibody (1:1,000) and then an AP‐linked rabbit anti‐goat IgG antibody (1:1,000) were added to the wells and allowed to incubate for 1 h at room temperature each; the plates were washed following each step. For development of the CT‐antibody complex, we utilized *p*‐nitrophenyl phosphate substrate solution (Invitrogen) according to the manufacturer's protocol. The color intensity in each well was measured at 405 nm in a plate reader. We estimated CT amounts in the samples by comparison to a standard curve.

### cAMP ELISA

HT‐29 cells were incubated for 4 h at 37°C with supernatants from *V. cholerae* cells grown in AKI media with or without mucin glycans for 3–4 h (OD_600_ = 0.5) at 37°C in anaerobic conditions. We filtered the supernatants through 0.22‐μm filters to remove all bacterial cells. Subsequently, we processed the HT‐29 cells according to the manufacturer's protocol to quantify cAMP levels in the cellular lysate using a cAMP activity assay kit (Abcam).

## Author contributions

**Benjamin X Wang:** Conceptualization; data curation; formal analysis; supervision; investigation; methodology; writing—original draft; writing—review and editing. **Julie Takagi:** Conceptualization; data curation; formal analysis; investigation; methodology; writing—original draft; writing—review and editing. **Abigail McShane:** Data curation; formal analysis; investigation. **Jin Hwan Park:** Data curation; formal analysis; investigation; writing—review and editing. **Kazuhiro Aoki:** Data curation; formal analysis. **Catherine Griffin:** Data curation; investigation. **Jennifer Teschler:** Investigation. **Giordan Kitts:** Investigation. **Giulietta Minzer:** Data curation; formal analysis; investigation. **Michael Tiemeyer:** Supervision; funding acquisition; investigation; writing—review and editing. **Rachel Hevey:** Data curation; formal analysis; supervision; funding acquisition; investigation; methodology; writing—review and editing. **Fitnat Yildiz:** Data curation; supervision; funding acquisition; investigation; methodology; project administration; writing—review and editing. **Katharina Ribbeck:** Conceptualization; supervision; funding acquisition; investigation; methodology; writing—review and editing.

## Disclosure and competing interests statement

The authors declare that they have no conflict of interest.

## Supporting information



Appendix

Expanded View Figures PDF

Table EV1

Table EV2

Dataset EV1

Source Data for Expanded View

PDF+

Source Data for Figure 1

## Data Availability

All raw mass spectrometry data related to mucin glycan profiles have been deposited at GlycoPost, ID GPST000254 (Watanabe *et al*, 2021), and can be accessed at https://glycopost.glycosmos.org/entry/GPST000254.1. All raw RNA‐seq data have been deposited to NIH GEO under accession GSE213598 (https://www.ncbi.nlm.nih.gov/geo/query/acc.cgi?acc=GSE213598).
