# Supplementary material for: Host‐derived O‐glycans inhibit toxigenic conversion by a virulence‐encoding phage in Vibrio cholerae
Source: EMBO J. 2022 Dec 12;42(3):e111562. doi: 10.15252/embj.2022111562 (PMC9890226; doi:10.15252/embj.2022111562)

Appendix Supplementary Information for Host-derived *O*-glycans inhibit toxigenic conversion by a virulence-encoding phage in *Vibrio cholerae*

Table of contents

Page 2-3: HPLC traces of synthesized glycan structures

HPLC Purity Analysis

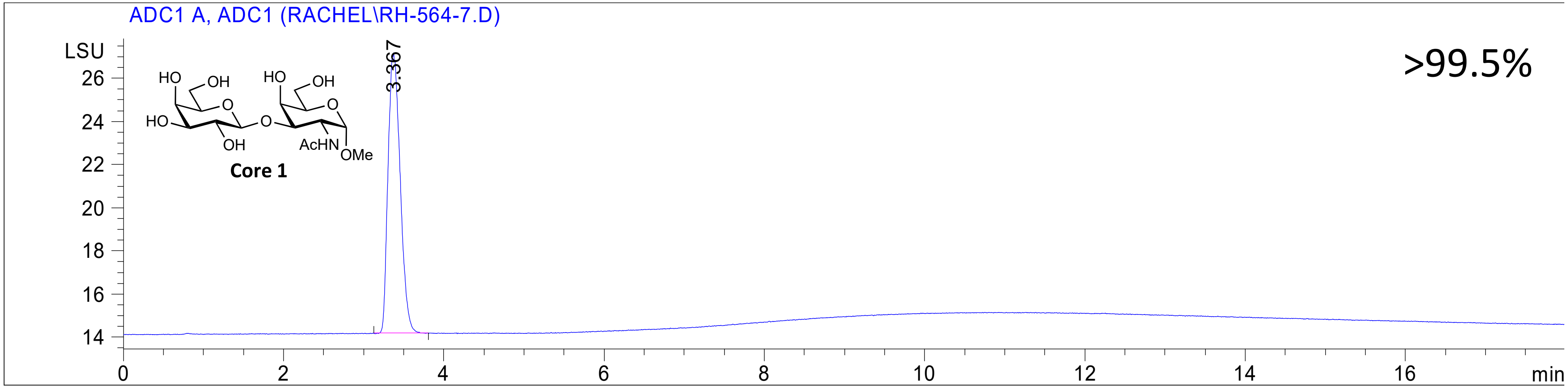

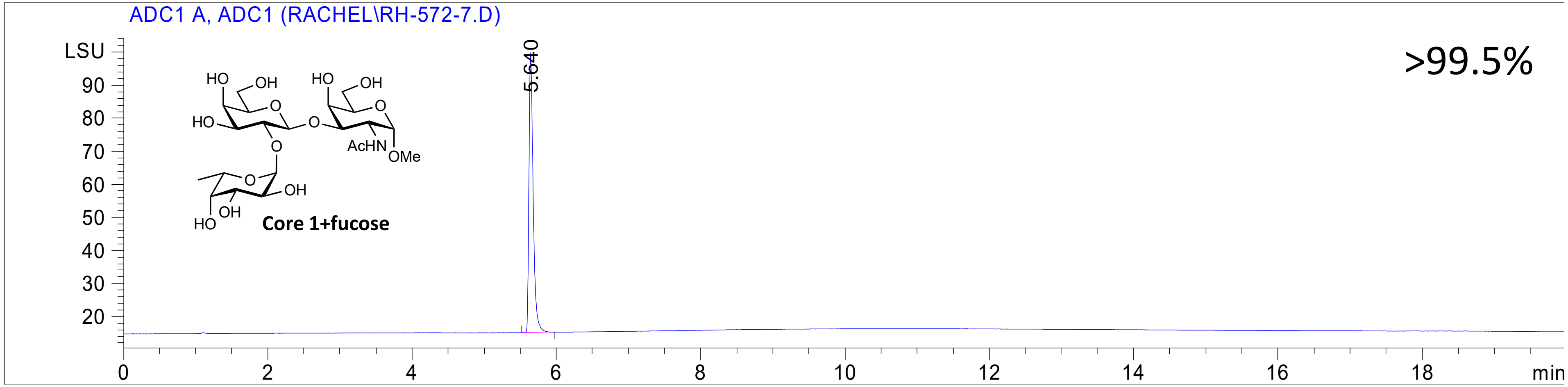

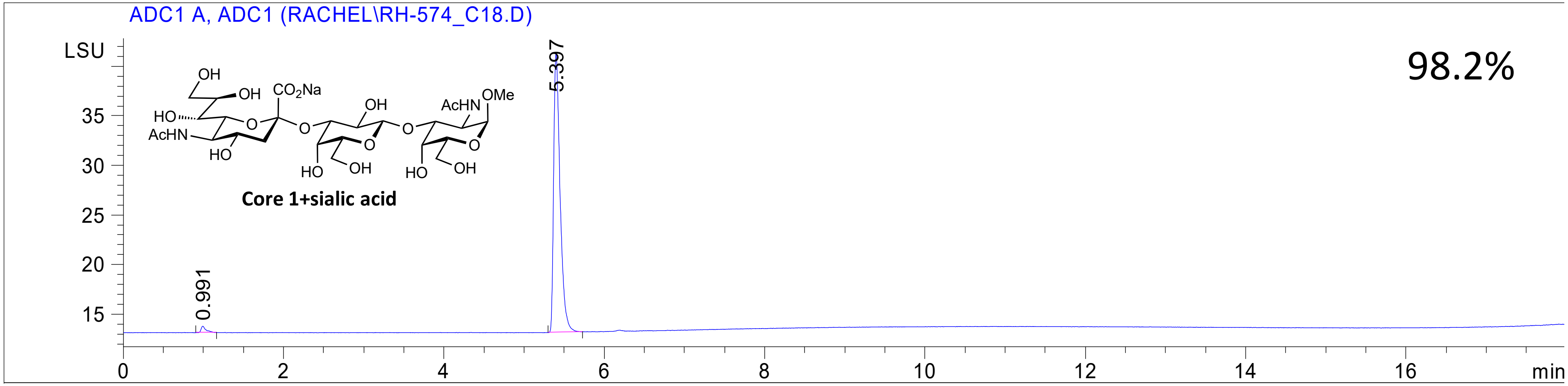

HPLC Purity Analysis

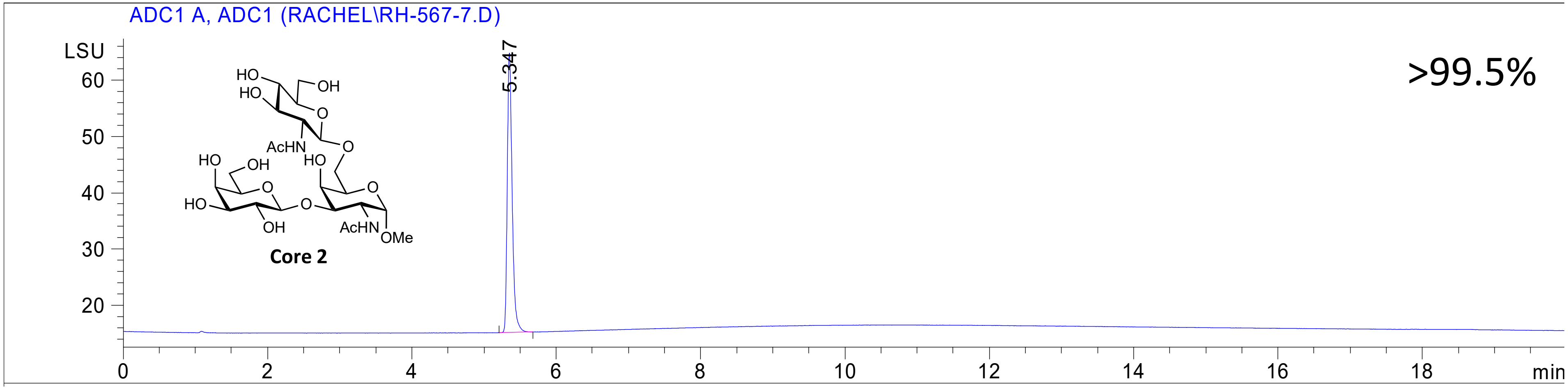

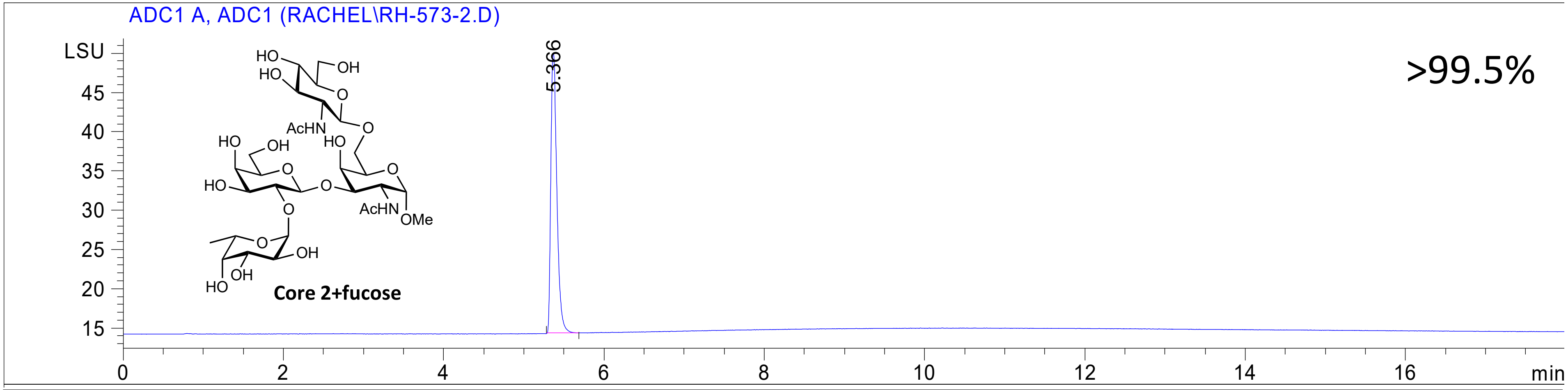

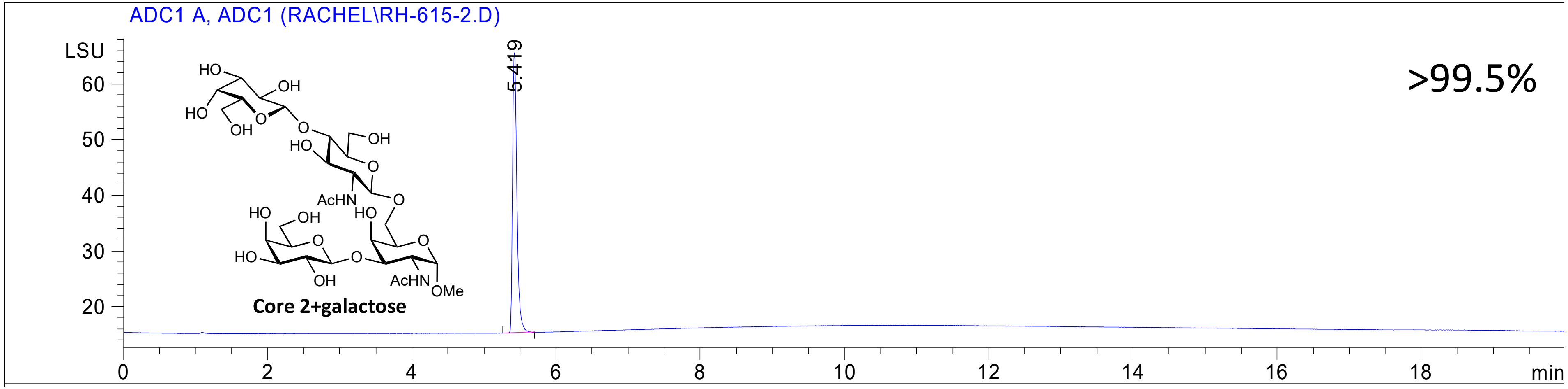

Supplement: Supplementary file 1 — Appendix [file EMBJ-42-e111562-s008.pdf]
