# Supplementary material for: Host‐derived O‐glycans inhibit toxigenic conversion by a virulence‐encoding phage in Vibrio cholerae
Source: EMBO J. 2022 Dec 12;42(3):e111562. doi: 10.15252/embj.2022111562 (PMC9890226; doi:10.15252/embj.2022111562)
Supplement: Supplementary file 2 — Expanded View Figures PDF [file EMBJ-42-e111562-s005.pdf]

Expanded View Figures

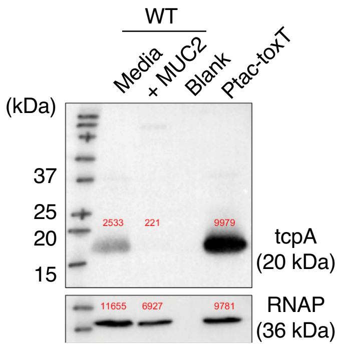

Figure EV1. Representative western blot image of TcpA from *V. cholerae* grown in the presence or absence of mucins, or in the IPTG-inducible *toxT* strain.

TcpA production in *V. cholerae* measured by Western blot of cells grown in the presence or absence of mucins. Cell pellets (normalized by total protein amounts) were subjected to Western blot analysis with anti-TcpA antibody. Band intensities are shown above each band. The image is derived from the same experiment as in Fig 1F.

Source data are available online for this figure.

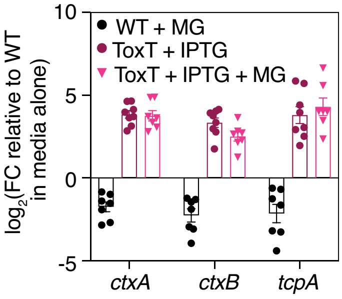

Figure EV2. Measurement of CTX-associated virulence gene expression in the IPTG-inducible *toxT* strain.

Induction of toxT with 1 mM of IPTG increases the expression of virulence genes relative to the wild-type [WT] strain. Gene expression was measured by qRT-PCR and normalized to a control gene (*gyrA*). Bars indicate mean ± standard error of the mean, with individual measurements of biological replicates ($n = 6$–8) shown. MG, mucin glycans.

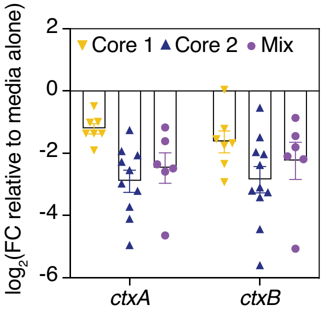

Figure EV3. The combination of Core 1 and Core 2 (mix) does not strongly alter the expression of cholera toxin- and TCP-encoding genes, compared with the Core 2 structure alone.

Gene expression was measured by qRT-PCR and normalized to a control gene (*gyrA*). Bars indicate mean ± SEM, with individual measurements of biological replicates ($n = 6$–11) shown. FC, fold change.

© 2022 The Authors
