# Supplementary figures and images for: Host‐derived O‐glycans inhibit toxigenic conversion by a virulence‐encoding phage in Vibrio cholerae
Source: EMBO J. 2022 Dec 12;42(3):e111562. doi: 10.15252/embj.2022111562 (PMC9890226; doi:10.15252/embj.2022111562)

150  
100  
75  
50  
37  
25  
20  
15  
10

For Fig. 1f, EV1

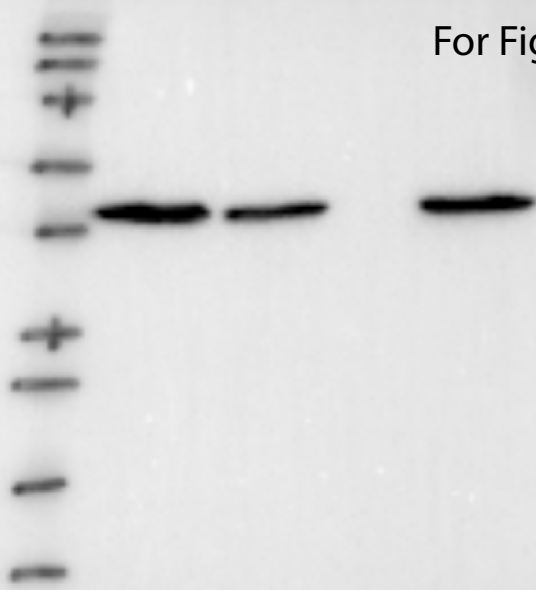

For Fig. 1f, EV1

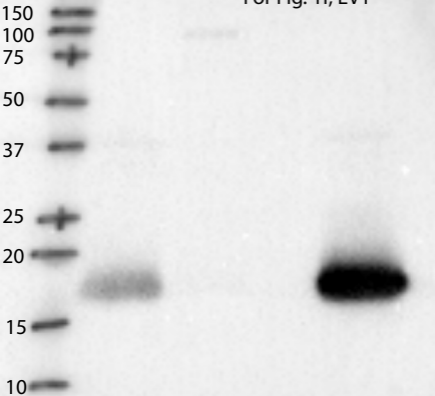

Supplement: Supplementary file 6 — Source Data for Expanded View [file EMBJ-42-e111562-s004.pdf]
