# Supplementary material for: Host‐derived O‐glycans inhibit toxigenic conversion by a virulence‐encoding phage in Vibrio cholerae
Source: EMBO J. 2022 Dec 12;42(3):e111562. doi: 10.15252/embj.2022111562 (PMC9890226; doi:10.15252/embj.2022111562)
Supplement: Supplementary file 9 [file EMBJ-42-e111562-s003.pdf]

THE EMBO JOURNAL

Host-derived O-glycans inhibit toxigenic conversion by a virulence-encoding phage in Vibrio cholerae

Benjamin Wang, Julie Takagi, Abigail McShane, Jin Park, Kazuhiro Aoki, Catherine Griffin, Jennifer Teschler, Giordan Kitts, Giulietta Minzer, Michael Tiemeyer, Rachel Hevey, Fitnat Yildiz, and Katharina Ribbeck
DOI: 10.15252/embj.2022111562

Corresponding author: Katharina Ribbeck (ribbeck@MIT.edu)

Review Timeline:

Submission Date:	29th Apr 22
Editorial Decision:	17th Jun 22
Revision Received:	26th Sep 22
Editorial Decision:	21st Oct 22
Revision Received:	25th Oct 22
Accepted:	4th Nov 22

Editor: Ieva Gailite

Transaction Report:

(Note: With the exception of the correction of typographical or spelling errors that could be a source of ambiguity, letters and reports are not edited. Depending on transfer agreements, referee reports obtained elsewhere may or may not be included in this compilation. Referee reports are anonymous unless the Referee chooses to sign their reports.)

1st Editorial Decision 17th Jun 2022

Thank you for submitting your manuscript for consideration by the EMBO Journal. We have now received four referee reports on your manuscript, which are included below for your information.

As you will see from the comments, reviewers 2 and 4 with expertise in mucin biology are more positive in their assessment, while also indicating a number of aspects that require further characterisation and clarification. However, reviewers 1 and 3 with expertise in V. cholerae biology indicate that more detailed insight into the molecular mode of O-glycan-mediated regulation of V. cholerae virulence and transformation would be required for publication here.

Based on the overall interest expressed in the referee reports and your willingness to engage in a major revision as described in the pre-decision consultation, I would like to invite you to address the comments of all reviewers in a revised version of the manuscript. I should add that it is The EMBO Journal policy to allow only a single major round of revision and that it is therefore important to resolve the main concerns at this stage.

We generally allow three months as standard revision time. As a matter of policy, competing manuscripts published during this period will not negatively impact on our assessment of the conceptual advance presented by your study. However, please contact me as soon as possible upon publication of any related work to discuss the appropriate course of action. Should you foresee a problem in meeting this three-month deadline, please let us know in advance and we may be able to grant an extension.

When preparing your letter of response to the referees' comments, please bear in mind that this will form part of the Review Process File and will therefore be available online to the community. For more details on our Transparent Editorial Process, please visit our website: https://www.embopress.org/page/journal/14602075/authorguide#transparentprocess. Please also see the attached instructions for further guidelines on preparation of the revised manuscript.

Please feel free to contact me if have any further questions regarding the revision. Thank you for the opportunity to consider your work for publication, and I look forward to receiving the revised manuscript.

Referee #1:

This ms characterizes the roles of mucins on CTX phage transduction/lysogenic conversion. Strong effects of mucin glycans are seen on expression of the major V. cholerae virulence genes, including ctxAB and the tcp operon. The authors conclude that this is happening via ToxT inhibition. However, the data are inconsistent with this conclusion as toxT transcription is very low in the

presence of the mucin products, indicating the effect is further upstream in the virulence cascade and not impacting ToxT activity. More work is really required to identify which transcription factor(s) is being impacted directly. It is likely that mucins are impacting ACTIVITY of either TcpP or ToxR and not abundance, and this should be tested directly.

Specific points:
1) Lines 11-12: It is inaccurate to say that CTX phage infects "natural members of the aquatic flora," as these are mostly non O1/O139 strains and lack the CTX phage receptor, TCP.
2) Line 44: V. cholerae inhabits fresh water bodies such as rivers and ponds as well and that is the source of infection. People do not drink brackish water.
3) The use of "hyper-virulent pathogen" repeatedly is a bit much and not really accurate.
4) Line 90: it is redundant to say "encodes for." You can either say encodes OR codes for.
5) The experiments in Fig 1 are lacking a negative control. Yes, they are being compared to media alone but there should be addition of some other substance as a negative control to compare with the mucin additions. Later some mucin monosaccharides and used and this would be appropriate here as well.
6) Line 113: should say expression levels of tcpA. This experiment would also be more convincing if actual TCP were assayed (agglutination assay, TcpA western, etc.)
7) Fig. 2b: a growth curve should be a semilog plot
8) Fig. 2d and 3f: toxT itself is being highly downregulated by mucin, which indicates that the effect on tcpA and ctx is not happening through toxT but through the regulators upstream of toxT that control its expression. This is therefore not a toxT-dependent mechanism as that implies a direct effect on ToxT activity. The clear hypothesis that springs from this is that TcpP or ToxR activity (and not abundance, since it is clear in Fig. 3f that they are not reduced in expression at the mRNA level) is being repressed by the mucin products and this should be tested directly.
9) The methods section is lacking information on who the bacterial cultures were grown in different media and this should be added. This is particularly important for using AKI, as there are multiple ways to induce virulence in AKI and whether bicarbonate is present is essential to know.
10) In Fig 3 there is an indication that ToxT activates tcpPH expression. This is incorrect. AphA and AphB activate tcpPH.

Referee #2:

Wang et al. observe that whole mucins and mucin glycans suppress the ability of CTφ to infect V. cholerae and mucin glycans downregulated the expression of the pilus-encoding tcpA gene. The authors then used RNAseq to look at changes in gene expression across the whole genome when V. cholerae was grown in the presence of mucin glycans. Again, this data showed TCP-encoding genes to be downregulated as well as both subunits of cholera toxin. This was shown to be the case in three media types, that monosaccharides were not individually responsible for the downregulation of these genes expression, and that it was dose-dependent. The authors also show that mucin glycans can reduce the cAMP levels in HT29 cells when they were exposed to V. cholerae. Finally, the authors were able to identify Core 2 as the specific mucin glycoprotein largely responsible for the effects described.

The work describes a likely important novel host-pathogen interaction. The data are very convincing and the claims about their meaning are well-balanced. I believe this would be of interest to a general audience. There are also a wide variety of techniques applied to build the evidence, from microbiology techniques to working with human intestinal cells, which is exceptional. The writing was very good, concise and clear.

A few comments:
1. The RNA-seq data would benefit from some kind of annotation to make it more accessible. A list plus details of upregulated and downregulated genes would be useful to the reader.
2. The upregulation was completed in rich-media types and I wonder how this would compare to what V. cholerae would be using as a nutrient source in the gut. Mucin has a lot of sialic acid, which V. cholerae has been shown to metabolise and I cannot see the GH33 in the RNA-seq data. Clarification around these issues would be good
3. The labels in Fig 2C are a bit crowded

Referee #3:

In this manuscript, Wang and coworkers studied the role of mucins and mucin-associated glycans on toxigenic conversion of Vibrio cholerae. Using various assays, they showed that purified mucin Muc2 or Muc5AC, or mucin glycans could prevent CTX phage transduction. Using RNA-Seq, and confirmed by qRT-PCR, they determined that Muc5AC and mucin glycans caused global change in V. cholerae gene expression. Specifically, expression of the genes in the ToxRS/TcpPH/ToxT regulon was repressed by mucins or mucin glycans but not by mucin monosaccharide. Using chemically synthesized mucin glycans, they determined that the Core 2 glycans are responsible for the observed change in gene repression.

In general, the research topic is important and interesting, which should be of interest to a general audience. However, there are places where experimental details were not provided, preventing proper interpretation of the results. Moreover, additional investigations are required to support the mechanism proposed by the authors in which ToxT is the key regulatory target. Here are my major comments on the manuscript:

1. In many experiments and assays, for example in the phage transduction assay shown Fig 1 and the qRT-PCR assay shown in Figs. 3 & 5, and the cAMP assay shown in Fig.4, it is unclear how much purified mucins, mucin glycans, or synthesized Core glycans were added to the cells. Were the amounts added physiologically relevant?

2. While there is a decrease in tcpA gene expression, it is important to show if V. cholerae still makes the Toxin-coregulated pili (TCP) in the presence of mucins or mucin glycans.

3. In fig. 1b, could the mucins just bind to the CTX phage and prevent the phage from interacting with the V. cholerae host? Similarly, could mucin glycans bind to TCP and block the binding of CTX to its receptor?

4. The down regulation of toxT transcript level was very modest (~1.5 fold, line 157, fig 3F), is there any change in the ToxT protein level that could explain the ~5-30 fold decrease in tcpA transcription (line 114) and 100-fold decrease in cholera toxin production (line 151)?

5. Similarly, how much ToxT was made when it is under control of a Ptac promoter with IPTG (Fig. 3G)? And when comparing WT with the Ptac-toxT strain without MG, is there a significant difference in ctxAB and tcpA gene expression? I supposed if these genes were expressed at very high levels in the Ptac-toxT strain, any difference in gene expression might not be observable due to the limits of qRT-PCR assays.

6. And if mucins or mucin glycans really target toxT gene expression and not through other mechanisms, how does it work? As shown by the authors (Fig 3F), the ToxT regulatory pathway is pretty well studied and many null and constitutive mutants have been characterized, it is important to know if TcpPH and ToxRS, or even other upstream regulatory component such as AphA, are involved.

Minor comments:
1. Fig 1 b and d have different headers (CTXΦ vs CTΦ).
2. Does Core 2 prevent CTX transduction?
3. In the supplementary table, only gene numbers are shown. It is unclear which gene belongs to which function category/pathway as shown in Fig. 2.
4. Based on the RNA-Seq data, there was a strong decrease in gene expression for genes in the ribonucleotide biosynthetic pathway, and an increase in biofilm gene expression when V. cholerae was treated with Muc5AC (fig 2). Could these genes affect CTX transduction as well?

Referee #4:

The study "Host-derived O-glycans inhibit toxigenic conversion by a virulence-encoding phage in Vibrio cholerae" by Wang and Takagi et al provide evidence that complex glycans on secreted gel-forming mucins repress virulence of V. cholerae, and important human pathogen. It is well known that gel-forming mucins like MUC2 in the intestine and MUC5AC in the stomach play important roles in the pathogenesis of infectious diseases, largely by forming a barrier to prevent or limit their invasion or by sometimes being subverted by pathogens to promote invasion. Either of these roles is influenced by the complex oligosaccharide repertoire that makes up the majority of the mucin mass, which are primarily of the O-GalNAc type of O-glycan. Thus, there is an important need to identify the biologic functions of O-glycans with specific pathogens to determine whether the interaction promotes or antagonizes infection. This succinct and elegant work by Wang and Takagi et al is part of a series of excellent studies by the Ribbeck group that directly addresses this question, showing here that O-glycans from native mucins extracted and purified from the gastrointestinal tracts of pigs have a direct role in repressing virulence of V. cholerae in vitro. Specifically, using a combination of virulence assays, microbial transcriptomics, glycomics, and functional studies, the authors show glycans work by inhibiting the toxigenic conversion of V. cholerae from its commensal to its pathogenic form by a two-fold mechanism: (i) blocking uptake (transduction) of the toxin-producing CTXφ phage by inhibiting expression of V. cholerae-encoded toxin co-regulated pilus (TCP), a major receptor for CTXφ; and (ii) repressing expression of CTXφ-encoded cholera toxin subunits ctxA and B as well as numerous other virulence factors (via transcriptomics and qPCR). The impact was functional because it prevented V. cholerae-induced deregulation of ion transport in HT-29 colon epithelial cells that leads to potentially deadly diarrhea in an in vivo situation. Gain-of-function approaches in toxigenic V. cholerae were consistent with the idea these repressive effects were attributed to glycan-dependent down-regulation of ToxT, a positive regulator of TCP and ctxA & B expression. Impressively, by synthesizing their own glycan library based on the glycan profiles from the native mucins, the authors were able to narrow down which among the many glycan structures was most impactful, identifying core 2 structures (vs core 1 and individual monosaccharides) were most potent to mediate this protective effect. While this is not the first study to show mucins can limit the virulence of V. cholerae (as pointed out by the authors already in the Discussion), this study is novel

in that it probes specifically how the glycans on these mucins mediate their protective effects. However, I have a few concerns that should be addressed:

MAJOR CONCERNS

1. In Fig 1b and e, and I the results sections, the authors show/describe data that native mucins from pig MUC2 and MUC5AC were able to downregulate the expression of the tcpA gene which encodes the receptor for the CTXφ phage. However, in the methods, the authors state only MUC5AC was used for the study and that only the pig stomachs were scraped (which expresses MUC5AC but not MUC2). What was the source of the MUC2 in this study? This is a critical question for the reasons outlined below.

2. Although the study is very well executed, it seems as though most of the work was done with glycans derived from the gastric MUC5AC and not intestinal MUC2. This is an important distinction because V. cholerae mediates its toxic effects in the intestine where MUC2 (not MUC5AC) is expressed and MUC2 harbors some important glycosylation distinctions vs the stomach: Among these include (i) a prominence of complex glycans derived from core 3 and core 4 structures. (ii) a much higher ratio of acidic (i.e sialylated and sulfated) structures vs mostly neutral gastric mucins. Because this study emphasizes the importance of specific core structures in the repression phenotype (Fig 5) it would be pertinent to include data on the impact of the more relevant (synthesized) core 3 and core 4 structures, including sialylated and sulfated structures on the virulence of V. cholerae.

3. The authors indicate they extracted "native" mucus from the pig tissues, and the associated methods indicate they collected the soluble portions of the scrapings (which were further processed and purified by Sepharose 4B column) and discarded the insoluble portion. This is in contrast to methods from Hansson and colleagues and Martens and colleagues who specifically isolate that portion of mucus that is insoluble in GuCl which is reduced and alkylated to break up the polymer and solubilize it. Is there a reason why the authors focused on the soluble form? Is there verification that they were actually working specifically with MUC5AC (and MUC2)?

4. Glycan analysis: (i) The samples were analyzed in positive ion mode, but this would likely limit the detection of sialylated and sulfated glycan variants. Is there a reason why positive ion was selected over negative ion mode, or why both were not analyzed? (ii) Regarding glycan annotation, the current structures were determined as follows in Methods "Mass peaks were manually annotated and assigned to a particular O-glycan composition based on known core structures" This seems a bit vague. Can the authors elaborate more clearly in Methods on how they arrived definitively at the structures in Fig 5? Was there a database or standards used? Was Tandem MS/MS used for any structures? This is important because the authors also detect O-Mannosylated glycans on the mucins, which is uncommon, so it would be interesting how this structure was determined.

5. The authors provide nice evidence the core 2 trisaccharide (Galb1,3(GlcNAcb1,6)GalNAcaSer/Thr) is the major mediator (vs core 1) on mucins that block virulence expression and that the addition of Fuc or Gal to this structure does not affect the virulence repression activity of this glycan. Since glycans are a mixture of core 1 and 2 types, it may be insightful to see if the addition of both core 1 and core 2 structures has a synergistic (or antagonist) interaction on virulence repression. This is in part due to the dramatic impact of the intact mucins on blocking transconductance in Fig 1b. Further, does core 2 extension impact its activity on V. cholerae?

MINOR CONCERNS

1. For the results section describing RNASeq analysis, the authors emphasize the down-regulated genes and make only cursory remarks about the genes that are upregulated, namely relating to biofilms. Since 57 genes were upregulated by the glycans, this should be elaborated on a bit more in the text.

2. While the in vitro results are very impressive, the translation to in vivo is complex. Consistent with the authors' conclusions, many people who acquire V . cholerae show minor symptoms. However, V. cholerae still routinely causes significant morbidity and mortality each year worldwide. How do the authors' conclusions relate to cases where people experience fulminant infections (i.e. severe toxin-induced diarrhea). This should be addressed more clearly in the discussion.

3. Figure 3b: It is noted that sample replicates range from 3 - 6. Presumably, all RNA was analyzed at the same time for the qPCR assays - was there sample loss? Related Figure 1e, there are only 2 replicates for the MUC2-induced tcpA repression, and significant variation in the ability of mucins to induce the downregulation (1 - 256 fold loss based on log calculation). What would account for this level of variation? The sample size should be at least 5 (as shown for mucin glycans (MG).

4. Lines 157 - 159. The authors state "To determine if mucin glycans depend on ToxT for the downregulation of CTXφ-related virulence genes, we incubated mucin glycans with a V. cholerae mutant in which the native promoter for toxT was replaced with an IPTG-inducible promoter...". This line is a bit misleading because it suggests ToxT is needed for the ability for the O-glycans to reduce virulence factor expression. Based on the glycan-dependent down-regulation of ToxT in the RNASeq data and the

IPTG studies which suggest ToxT inhibits O-glycan-dependent repression, it would be clearer to say "To determine the role of ToxT in mucin glycan dependent down-regulation of CTX-related virulence genes......"

Reviewer Response

We thank the referees for carefully reviewing our manuscript and their overall positive responses. We have addressed all comments and concerns and made the relevant modifications to the figures and text as detailed below in our point-by point response to the reviewers. We believe the suggestions made by the reviewers have substantially improved our manuscript. Comments by the reviewers are shown in black, and our response is shown in red.

Referee #1:

This ms characterizes the roles of mucins on CTX phage transduction/lysogenic conversion. Strong effects of mucin glycans are seen on expression of the major V. cholerae virulence genes, including ctxAB and the tcp operon. The authors conclude that this is happening via ToxT inhibition. However, the data are inconsistent with this conclusion as toxT transcription is very low in the presence of the mucin products, indicating the effect is further upstream in the virulence cascade and not impacting ToxT activity. More work is really required to identify which transcription factor(s) is being impacted directly. It is likely that mucins are impacting ACTIVITY of either TcpP or ToxR and not abundance, and this should be tested directly.

Specific points:
1) Lines 11-12: It is inaccurate to say that CTX phage infects "natural members of the aquatic flora," as these are mostly non O1/O139 strains and lack the CTX phage receptor, TCP.

We have removed this phrase.

2) Line 44: V. cholerae inhabits fresh water bodies such as rivers and ponds as well and that is the source of infection. People do not drink brackish water.

We have replaced “brackish water” with the reviewer’s suggestion of “water bodies such as rivers and ponds”.

3) The use of "hyper-virulent pathogen" repeatedly is a bit much and not really accurate.

We have adjusted this throughout the text.

4) Line 90: it is redundant to say "encodes for." You can either say encodes OR codes for.

We have adjusted this to “encodes”.

5) The experiments in Fig 1 are lacking a negative control. Yes, they are being compared to media alone but there should be addition of some other substance as a negative control to compare with the mucin additions. Later some mucin monosaccharides and used and this would be appropriate here as well.

We thank the reviewer for this important point and have included mucin monosaccharides as a negative control. We see that these monosaccharides do not inhibit CTX phage transduction (Fig 1D.) We have added these changes in the text as lines 110-114.

6) Line 113: should say expression levels of tcpA. This experiment would also be more convincing if actual TCP were assayed (agglutination assay, TcpA western, etc.)

We have adjusted the wording to say “expression levels of tcpA”. In addition, we have now performed a western blot using anti tcpA antibodies to find that TcpA protein levels decrease by ~10-fold in response to purified mucins (see new text from lines 126-130 and new Fig. 1f-g).

7) Fig. 2b: a growth curve should be a semilog plot

We have adjusted this.

8) Fig. 2d and 3f: toxT itself is being highly downregulated by mucin, which indicates that the effect on tcpA and ctx is not happening through toxT but through the regulators upstream of toxT that control its expression. This is therefore not a toxT-dependent mechanism as that implies a direct effect on ToxT activity. The clear hypothesis that springs from this is that TcpP or ToxR activity (and not abundance, since it is clear in Fig. 3f that they are not reduced in expression at the mRNA level) is being repressed by the mucin products and this should be tested directly.

We thank the reviewer for raising this point and apologize for the confusion. We did not mean to imply that mucins/glycans have a direct effect on ToxT activity, but that they exert their effects by inducing the downregulation of *toxT* gene expression. As the reviewer stated, this strongly suggests that mucins are inhibiting the activity of TcpP and/or ToxR, which are upstream regulators of *toxT.*

To test this hypothesis, we have followed the suggestion of Reviewer 3 and assayed the ability of mucins/glycans to downregulate virulence gene expression in a variety of mutants including single deletion mutants of *tcpP* and *toxR,* a double deletion of *tcpP/toxR,* and published single point mutations of TcpP including TcpC207S and TcpC218S that have previously been shown to ablate TcpP/H signaling (Yang et al., 2013 PNAS). We found that for each of these mutants, the ability of mucin glycans to downregulate *tcpA* and *ctxAB* was impaired or completely ablated, strongly suggesting that mucin glycans indeed interfere with the activity of these upstream regulators of *toxT* expression. We have included these results in the text as lines 205-216 and as Figure 4d-e.

9) The methods section is lacking information on who the bacterial cultures were grown in different media and this should be added. This is particularly important for using AKI, as there are multiple ways to induce virulence in AKI and whether bicarbonate is present is essential to know.

We have added in a citation in the methods section for our recipe (Iwanaga et al., 1986), which contains bicarbonate.

10) In Fig 3 there is an indication that ToxT activates tcpPH expression. This is incorrect. AphA and AphB activate tcpPH.

We have adjusted the figure accordingly.

Referee #2:

Wang et al. observe that whole mucins and mucin glycans suppress the ability of CTφ to infect V. cholerae and mucin glycans downregulated the expression of the pilus-encoding tcpA gene. The authors then used RNAseq to look at changes in gene expression across the whole genome when V. cholerae was grown in the presence of mucin glycans. Again, this data showed TCP-encoding genes to be downregulated as well as both subunits of cholera toxin. This was shown to be the case in three media types, that monosaccharides were not individually responsible for the downregulation of these genes expression, and that it was dose-dependent. The authors also show that mucin glycans can reduce the cAMP levels in HT29 cells when they were exposed to V. cholerae. Finally, the authors were able to identify Core 2 as the specific mucin glycoprotein largely responsible for the effects described.

The work describes a likely important novel host-pathogen interaction. The data are very convincing and the claims about their meaning are well-balanced. I believe this would be of interest to a general audience. There are also a wide variety of techniques applied to build the evidence, from microbiology techniques to working with human intestinal cells, which is exceptional. The writing was very good, concise and clear.

A few comments:
1. The RNA-seq data would benefit from some kind of annotation to make it more accessible. A list plus details of upregulated and downregulated genes would be useful to the reader.

We thank the reviewer for this comment and have added annotations to our RNA-seq data, which now includes gene names, description, GO terms, and known associated pathways.

2. The upregulation was completed in rich-media types and I wonder how this would compare to what V. cholerae would be using as a nutrient source in the gut. Mucin has a lot of sialic acid, which V. cholerae has been shown to metabolise and I cannot see the GH33 in the RNA-seq data. Clarification around these issues would be good

We do not see any significant gene expression changes in sialic acid-degrading enzymes in the RNA-seq data (ie, *nanH*), suggesting that *V. cholerae* is not metabolizing this sugar under our experimental conditions.

3. The labels in Fig 2C are a bit crowded

We have adjusted this.

Referee #3:

In this manuscript, Wang and coworkers studied the role of mucins and mucin-associated glycans on toxigenic conversion of Vibrio cholerae. Using various assays, they showed that purified mucin Muc2 or Muc5AC, or mucin glycans could prevent CTX phage transduction. Using RNA-Seq, and confirmed by qRT-PCR, they determined that Muc5AC and mucin glycans caused global change in V. cholerae gene expression. Specifically, expression of the genes in the ToxRS/TcpPH/ToxT regulon was repressed by mucins or mucin glycans but not by mucin monosaccharide. Using chemically synthesized mucin glycans, they determined that the Core 2 glycans are responsible for the observed change in gene repression.

In general, the research topic is important and interesting, which should be of interest to a general audience. However, there are places where experimental details were not provided, preventing proper interpretation of the results. Moreover, additional investigations are required to support the mechanism proposed by the authors in which ToxT is the key regulatory target. Here are my major comments on the manuscript:

1. In many experiments and assays, for example in the phage transduction assay shown Fig 1 and the qRT-PCR assay shown in Figs. 3 & 5, and the cAMP assay shown in Fig.4, it is unclear how much purified mucins, mucin glycans, or synthesized Core glycans were added to the cells. Were the amounts added physiologically relevant?

We have added some text to the methods section indicating that mucins were used at a concentration at 0.5% w/v and glycans were used at a concentration at 0.1% w/v, unless otherwise indicated in the figure legends (lines 349-352). These are within physiologically relevant concentrations within host mucosal tracts, as measured by previous papers (Bansil and Turner, 2018; Ridley and Thornton, 2018).

2. While there is a decrease in tcpA gene expression, it is important to show if V. cholerae still makes the Toxin-coregulated pili (TCP) in the presence of mucins or mucin glycans.

We thank the reviewer for this important point. We have now performed a western blot using anti tcpA antibodies to find that TcpA protein levels decrease by ~10-fold in response to purified mucins (see new text from lines 125-130 and new Fig. 1f-g).

3. In fig. 1b, could the mucins just bind to the CTX phage and prevent the phage from interacting with the V. cholerae host? Similarly, could mucin glycans bind to TCP and block the binding of CTX to its receptor?

We thank the reviewer for raising this interesting point. To test this hypothesis, we repeated the phage transduction assay using a strain where *toxT* expression is controlled from an IPTG-inducible promoter, in the presence or absence of mucin glycans. Under IPTG induction, this strain is unresponsive to mucin (Fig. 4b) while expressing high levels of *tcpA,* the phage receptor for CTX (EV Fig. 1-2). Thus, if mucin glycans are physically blocking the binding of CTX to its TcpA receptor through steric hinderance, then these glycans should still be able to prevent CTX phage transduction in the constitutive toxT strain, as there is both CTX phage and TcpA phage receptor present in the experiment. However, if mucin glycans block CTX phage

transduction through signaling pathways (ie, by downregulating *toxT* gene expression), then mucin glycans should no longer be able to block CTX phage transduction when *toxT* is constitutively expressed through IPTG induction.

Our new results show that mucin glycans no longer block CTX phage transduction in the constitutive *toxT* strain, suggesting that these glycans indeed inhibit CTX phage transduction through the downregulation of *toxT* gene expression and not through steric hinderance or binding to TCP. We have included these new results in the text as lines 197-204 and figure 4c.

4. The down regulation of toxT transcript level was very modest (~1.5 fold, line 157, fig 3F), is there any change in the ToxT protein level that could explain the ~5-30 fold decrease in tcpA transcription (line 114) and 100-fold decrease in cholera toxin production (line 151)?

We thank the reviewer for this question. We obtained anti-toxT antibodies from our collaborator (Dr. Karl Klose). However, we were unable to detect this protein even in the WT control, suggesting that the antibody is not sensitive enough to use under our particular experimental conditions. This is likely because we are severely limited by the amount of mucins/glycans we can use. For our assays, we normally use ~50-100 uL of vibrio+mucins/glycans; at a 0.1% w/v concentration, this requires ~50-100 ug of glycans for each experiment. As glycan purification is a labor-intensive process (see methods), we are only able to obtain ~1-2 mg of glycans from each prep, which takes ~2 months, making it very technically challenging to scale up our setup. Thus, under the technical limitations of working with mucins/glycans, we are unable to scale up our setup to high enough levels in order to detect adequate amounts of ToxT proteins by western blot using our obtained anti-ToxT antibody.

5. Similarly, how much ToxT was made when it is under control of a Ptac promoter with IPTG (Fig. 3G)? And when comparing WT with the Ptac-toxT strain without MG, is there a significant difference in ctxAB and tcpA gene expression? I supposed if these genes were expressed at very high levels in the Ptac-toxT strain, any difference in gene expression might not be observable due to the limits of qRT-PCR assays.

Using qRT-PCR, we observed that the levels of *ctxAB* and *tcpA* gene expression are ~15 fold higher in the IPTG-inducible *toxT* strain compared to the WT, where *toxT* expression is driven by its native promoter (see figure EV2). Based on our other qRT-PCR assays, in which we can easily detect >64-fold changes in gene expression (for example, figure 1e), we believe that our qPCR assay is sensitive enough to detect significant gene expression changes in the constitutive *toxT* strain, even with this elevated baseline.

6. And if mucins or mucin glycans really target toxT gene expression and not through other mechanisms, how does it work? As shown by the authors (Fig 3F), the ToxT regulatory pathway is pretty well studied and many null and constitutive mutants have been characterized, it is important to know if TcpPH and ToxRS, or even other upstream regulatory component such as AphA, are involved.

We thank the reviewer for raising this point. As the reviewer stated, our results suggest that mucins are inhibiting the activity of TcpP and/or ToxR, which are upstream regulators of *toxT*.

To test this hypothesis, we have assayed the ability of mucins/glycans to downregulate virulence gene expression in a variety of mutants including single deletion mutants of *tcpP* and *toxR,* a double deletion of *tcpP/toxR,* and published single point mutations of TcpP including TcpC207S and TcpP218S that have previously been shown to ablate TcpP/H signaling (Yang et al., 2013 PNAS). We found that for each of these mutants, the ability of mucin glycans to downregulate *tcpA* and *ctxAB* was impaired or ablated, strongly suggesting that mucin glycans indeed interfere with the activity of these upstream regulators of *toxT* expression. We have included these results in the text as lines 205-216 and as Figure 4d-e.

Minor comments:
1. Fig 1 b and d have different headers (CTXΦ vs CTΦ).

We have adjusted this.

2. Does Core 2 prevent CTX transduction?

We thank the reviewer for this suggestion and have now seen that the purified core 2 structure decreases CTX phage transduction by ~50%. However, we do not yet have enough replicates to include this data in the main manuscript (pending the arrival of new core-2 synthesized glycans), so we have included a figure below for the reviewer to view:

Figure for reviewers removed

3. In the supplementary table, only gene numbers are shown. It is unclear which gene belongs to which function category/pathway as shown in Fig. 2.

We have added annotations to the RNA-seq tables, including gene names, descriptions, GO terms, and associated pathways.

4. Based on the RNA-Seq data, there was a strong decrease in gene expression for genes in the ribonucleotide biosynthetic pathway, and an increase in biofilm gene expression when V. cholerae was treated with Muc5AC (fig 2). Could these genes affect CTX transduction as well?

We would expect an increase in biofilm gene expression/formation to correlate with an increase in CTX transduction (Gallego-Hernandez et. al., 2020). Interestingly, our data suggests that mucins increase biofilm gene expression while simultaneously decreasing CTX gene expression, suggesting that mucins “break” the virulence circuits that others have found. We have expanded on this point in the discussion (see lines 270-280). There is no clear relationship between ribonucleotide biosynthesis and CTX transduction that we could find in the literature.

Referee #4:

The study "Host-derived O-glycans inhibit toxigenic conversion by a virulence-encoding phage in Vibrio cholerae" by Wang and Takagi et al provide evidence that complex glycans on secreted gel-forming mucins repress virulence of V. cholerae, and important human pathogen. It is well known that gel-forming mucins like MUC2 in the intestine and MUC5AC in the stomach play important roles in the pathogenesis of infectious diseases, largely by forming a barrier to prevent or limit their invasion or by sometimes being subverted by pathogens to promote invasion. Either of these roles is influenced by the complex oligosaccharide repertoire that makes up the majority of the mucin mass, which are primarily of the O-GalNAc type of O-glycan. Thus, there is an important need to identify the biologic functions of O-glycans with specific pathogens to determine whether the interaction promotes or antagonizes infection. This succinct and elegant work by Wang and Takagi et al is part of a series of excellent studies by the Ribbeck group that directly addresses this question, showing here that O-glycans from native mucins extracted and purified from the gastrointestinal tracts of pigs have a direct role in repressing virulence of V. cholerae in vitro. Specifically, using a combination of virulence assays, microbial transcriptomics, glycomics, and functional studies, the authors show glycans work by inhibiting the toxigenic conversion of V. cholerae from its commensal to its pathogenic form by a two-fold mechanism: (i) blocking uptake (transduction) of the toxin-producing CTXφ phage by inhibiting expression of V. cholerae-encoded toxin co-regulated pilus (TCP), a major receptor for CTXφ; and (ii) repressing expression of CTXφ-encoded cholera toxin subunits ctxA and B as well as numerous other virulence factors (via transcriptomics and qPCR). The impact was functional because it prevented V. cholerae-induced deregulation of ion transport in HT-29 colon epithelial cells that leads to potentially deadly diarrhea in an in vivo situation. Gain-of-function approaches in toxigenic V. cholerae were consistent with the idea these repressive effects were attributed to glycan-dependent down-regulation of ToxT, a positive regulator of TCP and ctxA & B expression. Impressively, by synthesizing their own glycan library based on the glycan profiles from the native mucins, the authors were able to narrow down which among the many glycan structures was most impactful, identifying core 2 structures (vs core 1 and individual monosaccharides) were most potent to mediate this protective effect. While this is not the first study to show mucins can limit the virulence of V. cholerae (as pointed out by the authors already in the Discussion), this study is novel in that it probes specifically how the glycans on these mucins mediate their protective effects. However, I have a few concerns that should be addressed:

MAJOR CONCERNS

1. In Fig 1b and e, and I the results sections, the authors show/describe data that native mucins from pig MUC2 and MUC5AC were able to downregulate the expression of the tcpA gene which encodes the receptor for the CTXφ phage. However, in the methods, the authors state only MUC5AC was used for the study and that only the pig stomachs were scraped (which expresses MUC5AC but not MUC2). What was the source of the MUC2 in this study? This is a critical question for the reasons outlined below.

Our MUC2 was obtained from fresh pig intestines which we now describe in more details in the methods.

2. Although the study is very well executed, it seems as though most of the work was done with glycans derived from the gastric MUC5AC and not intestinal MUC2. This is an important distinction because V. cholerae mediates its toxic effects in the intestine where MUC2 (not MUC5AC) is expressed and MUC2 harbors some important glycosylation distinctions vs the stomach: Among these include (i) a prominence of complex glycans derived from core 3 and core 4 structures. (ii) a much higher ratio of acidic (i.e sialylated and sulfated) structures vs mostly neutral gastric mucins. Because this study emphasizes the importance of specific core structures in the repression phenotype (Fig 5) it would be pertinent to include data on the impact of the more relevant (synthesized) core 3 and core 4 structures, including sialylated and sulfated structures on the virulence of V. cholerae.

Most of our study utilizes MUC5AC glycans because these glycans are much more easily obtainable at the scale that we need for our functional and transcriptional assays. In addition, our previous publications (Takagi et. al., 2022) show that our MUC5AC and MUC2 glycans from the porcine gastrointestinal tract have substantial overlap.

Consistent with the literature, MUC2 glycans had had higher prevalence of sialylated and Core 3 structures compared to MUC5AC. Sialylated structures compose 19% and 2.6% of the glycan pool in MUC2 and MUC5AC respectively. Additionally, we observed that 6.3% and 2.5% of the glycan pool is composed of Core 3 structures in MUC2 and MUC5AC respectively. We did not observe any Core 4 structures.

For testing of the individual mucin glycan structures, we are limited by accessibility and scope of the current synthetic methods. It is well recognized among glycochemists that the synthesis and purification of complex glycans takes months for smaller structures (e.g., trisaccharides) and years for larger ones (e.g., hexasaccharides), also depending on complexity, due to the complicated protection-deprotection strategies that need to be developed in order to obtain single pure structures with the desired glycosidic linkages at the correct subunit positions and with correct stereochemistry. The negatively charged sialylated and sulfated glycans are particularly challenging, due to their inherent instability (which limits the range of protection-deprotection strategies that can be used) and significantly more challenging purifications. In fact, the current synthetic method reported within to synthesize the mucin O-glycan structures would not be compatible with sulfated structures, and therefore further chemical method development would be required before gaining access to this class of glycans. We have been successful with the synthesis of core 1-Neu5Ac glycans, although larger sialylated glycans are also beyond the scope of the current method and in future work potentially a mixed chemo-enzymatic approach could be envisaged. With regards to the core 3 and core 4 glycans, we have not yet expanded the scope of the synthetic method to include these structures, but this is ongoing work and given the time required we also consider to be beyond the scope of the current publication.

3. The authors indicate they extracted "native" mucus from the pig tissues, and the associated methods indicate they collected the soluble portions of the scrapings (which were further processed and purified by Sepharose 4B column) and discarded the insoluble portion. This is in contrast to methods from Hansson and colleagues and Martens and colleagues who specifically isolate that portion of mucus that is insoluble in GuCl which is reduced and alkylated to break up

the polymer and solubilize it. Is there a reason why the authors focused on the soluble form? Is there verification that they were actually working specifically with MUC5AC (and MUC2)?

Our MUC2 and MUC5Ac as obtained from pig intestines and stomachs respectively using size exclusion chromatography, which preserves the native mucin structure. The use of chaotropic and denaturing solvents, such as guanidium chloride, results in mucins that are about 20 times more permeable than native mucus (Thornton and Sheehan, 2003). Our purification which utilizes nonchaotropic agents preserves the gel-forming structure of mucus (Wagner and Wheeler et. al., 2018; Wagner et. al. 2017).

Mass spectrometry is routinely used to monitor the composition of purified mucin extracts. This type of analysis has shown that mucin extracts purified from porcine stomach mucus, for example, are composed predominantly of MUC5AC, with small quantities of MUC2, MUC5B and MUC6, as well as histones, actin and albumin (Caldara et. al. 2012; Lieleg et. al. 2012).

4. Glycan analysis: (i) The samples were analyzed in positive ion mode, but this would likely limit the detection of sialylated and sulfated glycan variants. Is there a reason why positive ion was selected over negative ion mode, or why both were not analyzed? (ii) Regarding glycan annotation, the current structures were determined as follows in Methods "Mass peaks were manually annotated and assigned to a particular O-glycan composition based on known core structures" This seems a bit vague. Can the authors elaborate more clearly in Methods on how they arrived definitively at the structures in Fig 5? Was there a database or standards used? Was Tandem MS/MS used for any structures? This is important because the authors also detect O-Mannosylated glycans on the mucins, which is uncommon, so it would be interesting how this structure was determined.

We apologize for the confusion and have clarified our methods. The incorrect text has been removed and the correct method has now been added in a new section entitled "Structural analysis of mucin O-glycans." The reviewer is correct that positive mode analysis of non-derivatized glycans would have severely underestimated the representation of sialylated glycans. In the new methods text, we describe that the glycans were permethylated prior to analysis, neutralizing the sialylated glycans and enhancing the sensitivity of detection and the degree of structural determination obtainable by MS/MS and Msn fragmentation. The O-Man glycans were assigned based on the presence of a reducing terminal Hex with an associated HexNAc. The only other type of O-glycan with a reducing terminal Hex residue is the O-Glc found on thrombospondin and EGF repeats. However, in this case, the Hex is associated with a Xyl residue not a HexNAc, each possessing a clearly distinguishable m/z profile. Overall, we have clarified the methods for this in lines 416-433 in the methods section.

5. The authors provide nice evidence the core 2 trisaccharide (Galb1,3(GlcNAcb1,6)GalNAcaSer/Thr) is the major mediator (vs core 1) on mucins that block virulence expression and that the addition of Fuc or Gal to this structure does not affect the virulence repression activity of this glycan. Since glycans are a mixture of core 1 and 2 types, it may be insightful to see if the addition of both core 1 and core 2 structures has a synergistic (or antagonist) interaction on virulence repression. This is in part due to the dramatic impact of the intact mucins on blocking transconductance in Fig 1b. Further, does core 2 extension impact its activity on V. cholerae?

We thank the reviewer for this suggestion and have tested whether a mixture of core 1 and 2 glycans further decrease virulence gene expression. However, we found that core1+core2 decreased CTX gene expression to the same degree as core 2 alone, further suggesting that the core 2 structure is primarily involved in this virulence attenuation (Fig. EV3).

MINOR CONCERNS

1. For the results section describing RNASeq analysis, the authors emphasize the down-regulated genes and make only cursory remarks about the genes that are upregulated, namely relating to biofilms. Since 57 genes were upregulated by the glycans, this should be elaborated on a bit more in the text.

We have expanded this discussion in the text in lines 270-280. Interestingly, we would expect an increase in biofilm gene expression/formation to correlate with an increase in CTX transduction (Gallego-Hernandez et. al., 2020). However, our data suggests that mucins increase biofilm gene expression while simultaneously decreasing CTX gene expression, suggesting that mucins “break” the virulence circuits that others have found.

2. While the in vitro results are very impressive, the translation to in vivo is complex. Consistent with the authors' conclusions, many people who acquire V . cholerae show minor symptoms. However, V. cholerae still routinely causes significant morbidity and mortality each year worldwide. How do the authors' conclusions relate to cases where people experience fulminant infections (i.e. severe toxin-induced diarrhea). This should be addressed more clearly in the discussion.

We have added a few sentences to the discussion (see lines 310-315).

3. Figure 3b: It is noted that sample replicates range from 3 - 6. Presumably, all RNA was analyzed at the same time for the qPCR assays - was there sample loss? Related Figure 1e, there are only 2 replicates for the MUC2-induced tcpA repression, and significant variation in the ability of mucins to induce the downregulation (1 - 256 fold loss based on log calculation). What would account for this level of variation? The sample size should be at least 5 (as shown for mucin glycans (MG).

We have repeated these experiments and added in the new data points to figure 1e.

4. Lines 157 - 159. The authors state "To determine if mucin glycans depend on ToxT for the downregulation of CTXφ-related virulence genes, we incubated mucin glycans with a V. cholerae mutant in which the native promoter for toxT was replaced with an IPTG-inducible promoter...". This line is a bit misleading because it suggests ToxT is needed for the ability for the O-glycans to reduce virulence factor expression. Based on the glycan-dependent down-regulation of ToxT in the RNASeq data and the IPTG studies which suggest ToxT inhibits O-glycan-dependent repression, it would be clearer to say "To determine the role of ToxT in mucin glycan dependent down-regulation of CTX-related virulence genes......"

We thank the reviewer and have adjusted this in the text.

Thank you for submitting a revised version of your manuscript. Your study has now been seen by three of the original referees, who find that their previous concerns have been addressed and now recommend publication of the manuscript. There remain only a couple of minor editorial points that have to be addressed before I can extend formal acceptance of the manuscript.

Please let me know if you have any further questions regarding any of these points. You can use the link below to upload the revised files.

Thank you again for giving us the chance to consider your manuscript for The EMBO Journal. I look forward to receiving the final version.

Referee #1:

The authors have addressed all of my concerns in the manuscript, and performed the suggested additional experiments. I have no further concerns.

Referee #3:

The authors have addressed my previous concerns.

Referee #4:

The authors have sufficiently addressed my concerns. Congratulations to Ribbeck and colleagues on this technically and conceptually innovative study.

The authors addressed the remaining editorial issues.

Editor accepted the revised manuscript.

EMBO Press Author Checklist

Corresponding Author Name: Katharina Ribbeck	
Journal Submitted to: EMBO	
Manuscript Number: EMBOJ-2022-111562	

USEFUL LINKS FOR COMPLETING THIS FORM
The EMBO Journal - Author Guidelines
EMBO Reports - Author Guidelines
Molecular Systems Biology - Author Guidelines
EMBO Molecular Medicine - Author Guidelines

Reporting Checklist for Life Science Articles (updated January 2022)
This checklist is adapted from Materials Design Analysis Reporting (MDAR) Checklist for Authors. MDAR establishes a minimum set of requirements in transparent reporting in the life sciences (see Statement of Task: 10.31222/osf.io/9sm4x). Please follow the journal's guidelines in preparing your manuscript.
Please note that a copy of this checklist will be published alongside your article.

Abridged guidelines for figures
1. Data
The data shown in figures should satisfy the following conditions:
- the data were obtained and processed according to the field's best practice and are presented to reflect the results of the experiments in an accurate and unbiased manner.
- ideally, figure panels should include only measurements that are directly comparable to each other and obtained with the same assay.
- plots include clearly labeled error bars for independent experiments and sample sizes. Unless justified, error bars should not be shown for technical replicates.
- if n<5, the individual data points from each experiment should be plotted. Any statistical test employed should be justified.
- Source Data should be included to report the data underlying figures according to the guidelines set out in the authorship guidelines on Data Presentation.

2. Captions
Each figure caption should contain the following information, for each panel where they are relevant:
- a specification of the experimental system investigated (eg cell line, species name).
- the assay(s) and method(s) used to carry out the reported observations and measurements.
- an explicit mention of the biological and chemical entity(ies) that are being measured.
- an explicit mention of the biological and chemical entity(ies) that are altered/varied/perturbed in a controlled manner.
- the exact sample size (n) for each experimental group/condition, given as a number, not a range;
- a description of the sample collection allowing the reader to understand whether the samples represent technical or biological replicates (including how many animals, litters, cultures, etc.).
- a statement of how many times the experiment shown was independently replicated in the laboratory.
- definitions of statistical methods and measures:
  - common tests, such as t-test (please specify whether paired vs. unpaired), simple χ^2 tests, Wilcoxon and Mann-Whitney tests, can be unambiguously identified by name only, but more complex techniques should be described in the methods section;
  - are tests one-sided or two-sided?
  - are there adjustments for multiple comparisons?
  - exact statistical test results, e.g., P values = x but not P values < x;
  - definition of 'center values' as median or average;
  - definition of error bars as s.d. or s.e.m.

Please complete ALL of the questions below.
Select "Not Applicable" only when the requested information is not relevant for your study.

Materials

Newly Created Materials	Information included in the manuscript?	In which section is the information available? (Reagents and Tools Table, Materials and Methods, Figures, Data Availability Section)
New materials and reagents need to be available; do any restrictions apply?	Not Applicable	

Antibodies	Information included in the manuscript?	In which section is the information available? (Reagents and Tools Table, Materials and Methods, Figures, Data Availability Section)
For **antibodies** provide the following information:<br>- Commercial antibodies: RRID (if possible) or supplier name, catalogue number and or/clone number<br>- Non-commercial: RRID or citation	Yes	Materials and methods

DNA and RNA sequences	Information included in the manuscript?	In which section is the information available? (Reagents and Tools Table, Materials and Methods, Figures, Data Availability Section)
**Short novel DNA or RNA including primers, probes**: provide the sequences.	Yes	Table EV2

Cell materials	Information included in the manuscript?	In which section is the information available? (Reagents and Tools Table, Materials and Methods, Figures, Data Availability Section)
**Cell lines:** Provide species information, strain. Provide accession number in repository **OR** supplier name, catalog number, clone number, and/**OR** RRID.	Yes	Materials and methods
**Primary cultures:** Provide species, strain, sex of origin, genetic modification status.	Not Applicable	
Report if the cell lines were recently **authenticated** (e.g., by STR profiling) and tested for mycoplasma contamination.	Not Applicable	

Experimental animals	Information included in the manuscript?	In which section is the information available? (Reagents and Tools Table, Materials and Methods, Figures, Data Availability Section)
**Laboratory animals or Model organisms:** Provide species, strain, sex, age, genetic modification status. Provide accession number in repository **OR** supplier name, catalog number, clone number, **OR** RRID.	Not Applicable	
**Animal observed in or captured from the field:** Provide species, sex, and age where possible**.**	Not Applicable	
Please detail **housing and husbandry conditions**.	Not Applicable	

Plants and microbes	Information included in the manuscript?	In which section is the information available? (Reagents and Tools Table, Materials and Methods, Figures, Data Availability Section)
**Plants:** provide species and strain, ecotype and cultivar where relevant, unique accession number if available, and source (including location for collected wild specimens).	Not Applicable	
**Microbes:** provide species and strain, unique accession number if available, and source.	Yes	Table EV1

Human research participants	Information included in the manuscript?	In which section is the information available? (Reagents and Tools Table, Materials and Methods, Figures, Data Availability Section)
If collected and within the bounds of privacy constraints report on age, sex and gender or ethnicity for all study participants.	Not Applicable	

Core facilities	Information included in the manuscript?	In which section is the information available? (Reagents and Tools Table, Materials and Methods, Figures, Data Availability Section)
If your work benefited from core facilities, was their service mentioned in the acknowledgments section?	Yes	Acknowledgments section and methods

Design

Study protocol	Information included in the manuscript?	In which section is the information available? (Reagents and Tools Table, Materials and Methods, Figures, Data Availability Section)
If study protocol has been **pre-registered, provide DOI in the manuscript**. For clinical trials, provide the trial registration number **OR** cite DOI.	Not Applicable	
Report the **clinical trial registration number** (at ClinicalTrials.gov or equivalent), where applicable.	Not Applicable	

Laboratory protocol	Information included in the manuscript?	In which section is the information available? (Reagents and Tools Table, Materials and Methods, Figures, Data Availability Section)
Provide DOI OR other citation details if **external detailed step-by-step protocols** are available.	Not Applicable	

Experimental study design and statistics	Information included in the manuscript?	In which section is the information available? (Reagents and Tools Table, Materials and Methods, Figures, Data Availability Section)
Include a statement about **sample size** estimate even if no statistical methods were used.	Yes	Figure legends
Were any steps taken to minimize the effects of subjective bias when allocating animals/samples to treatment (e.g. **randomization procedure**)? If yes, have they been described?	Not Applicable	
Include a statement about **blinding** even if no blinding was done.	Not Applicable	
Describe **inclusion/exclusion criteria** if samples or animals were excluded from the analysis. Were the criteria pre-established?<br><br>If sample or data points were omitted from analysis, report if this was due to attrition or intentional exclusion and provide justification.	Not Applicable	
For every figure, are **statistical tests** justified as appropriate? Do the data meet the assumptions of the tests (e.g., normal distribution)? Describe any methods used to assess it. Is there an estimate of variation within each group of data? Is the variance similar between the groups that are being statistically compared?	Not Applicable	

Sample definition and in-laboratory replication	Information included in the manuscript?	In which section is the information available? (Reagents and Tools Table, Materials and Methods, Figures, Data Availability Section)
In the figure legends: state number of times the experiment was **replicated** in laboratory.	Yes	Included in figure legends
In the figure legends: define whether data describe **technical or biological replicates**.	Yes	Included in figure legends

Ethics

Ethics	Information included in the manuscript?	In which section is the information available? (Reagents and Tools Table, Materials and Methods, Figures, Data Availability Section)
Studies involving **human participants**: State details of **authority granting ethics approval** (IRB or equivalent committee(s), provide reference number for approval.	Not Applicable	
Studies involving **human participants**: Include a statement confirming that **informed consent** was obtained from all subjects and that the experiments conformed to the principles set out in the WMA Declaration of Helsinki and the Department of Health and Human Services Belmont Report.	Not Applicable	
Studies involving **human participants:** For publication of **patient photos**, include a statement confirming that consent to publish was obtained.	Not Applicable	
Studies involving experimental **animals**: State details of **authority granting ethics approva**l (IRB or equivalent committee(s), provide reference number for approval. Include a statement of compliance with ethical regulations.	Not Applicable	
Studies involving **specimen and field samples:** State if relevant **permits** obtained, provide details of authority approving study; if none were required, explain why.	Not Applicable	

Dual Use Research of Concern (DURC)	Information included in the manuscript?	In which section is the information available? (Reagents and Tools Table, Materials and Methods, Figures, Data Availability Section)
Could your study fall under dual use research restrictions? Please check biosecurity documents and list of **select agents and toxins** (CDC): https://www.selectagents.gov/sat/list.htm	Not Applicable	
If you used a select agent, is the security level of the lab appropriate and reported in the manuscript?	Not Applicable	
If a study is subject to dual use research of concern regulations, is the name of the **authority granting approval and reference number** for the regulatory approval provided in the manuscript?	Not Applicable	

Reporting

The MDAR framework recommends adoption of discipline-specific guidelines, established and endorsed through community initiatives. Journals have their own policy about requiring specific guidelines and recommendations to complement MDAR.

Adherence to community standards	Information included in the manuscript?	In which section is the information available? (Reagents and Tools Table, Materials and Methods, Figures, Data Availability Section)
State if relevant guidelines or checklists (e.g., **ICMJE, MIBBI, ARRIVE, PRISMA**) have been followed or provided.	Not Applicable	
For **tumor marker prognostic studies,** we recommend that you follow the **REMARK** reporting guidelines (see link list at top right). See author guidelines, under 'Reporting Guidelines'. Please confirm you have followed these guidelines.	Not Applicable	
For **phase II and III randomized controlled trials**, please refer to the **CONSORT** flow diagram (see link list at top right) and submit the CONSORT checklist (see link list at top right) with your submission. See author guidelines, under 'Reporting Guidelines'. Please confirm you have submitted this list.	Not Applicable	

Data Availability

Data availability	Information included in the manuscript?	In which section is the information available? (Reagents and Tools Table, Materials and Methods, Figures, Data Availability Section)
Have **primary datasets** been deposited according to the journal's guidelines (see 'Data Deposition' section) and the respective accession numbers provided in the Data Availability Section?	Yes	Data Avaliability Section
Were **human clinical and genomic datasets** deposited in a public access-controlled repository in accordance to ethical obligations to the patients and to the applicable consent agreement?	Not Applicable	
Are **computational models** that are central and integral to a study available without restrictions in a machine-readable form? Were the relevant accession numbers or links provided?	Not Applicable	
If publicly available data were reused, provide the respective **data citations in the reference list**.	Not Applicable	
